# Alzheimer’s disease associated isoforms of human CD33 distinctively modulate microglial cell responses in 5XFAD mice

**DOI:** 10.1186/s13024-024-00734-8

**Published:** 2024-05-27

**Authors:** Ghazaleh Eskandari-Sedighi, Madeline Crichton, Sameera Zia, Erik Gomez-Cardona, Leonardo M. Cortez, Zain H. Patel, Kei Takahashi-Yamashiro, Chris D. St. Laurent, Gaurav Sidhu, Susmita Sarkar, Vivian Aghanya, Valerie L. Sim, Qiumin Tan, Olivier Julien, Jason R. Plemel, Matthew S. Macauley

**Affiliations:** 1 Department of Chemistry, University of Alberta, Edmonton, Canada; 2 Division of Neurology, Department of Medicine, University of Alberta, Edmonton, Canada; 3 Department of Biochemistry, University of Alberta, Edmonton, Canada; 4 Centre for Prions and Protein Folding Diseases, University of Alberta, Edmonton, Canada; 5 Neuroscience and Mental Health Institute, University of Alberta, Edmonton, Canada; 6 Department of Cell Biology, University of Alberta, Edmonton, Canada; 7 Department of Medical Microbiology and Immunology, University of Alberta, Edmonton, Canada

**Keywords:** Alzheimer’s disease, CD33, Microglia, Plaque compaction, Siglec, Amyloid-beta

## Abstract

Microglia play diverse pathophysiological roles in Alzheimer’s disease (AD), with genetic susceptibility factors skewing microglial cell function to influence AD risk. CD33 is an immunomodulatory receptor associated with AD susceptibility through a single nucleotide polymorphism that modulates mRNA splicing, skewing protein expression from a long protein isoform (CD33M) to a short isoform (CD33m). Understanding how human CD33 isoforms differentially impact microglial cell function in vivo has been challenging due to functional divergence of CD33 between mice and humans. We address this challenge by studying transgenic mice expressing either of the human CD33 isoforms crossed with the 5XFAD mouse model of amyloidosis and find that human CD33 isoforms have opposing effects on the response of microglia to amyloid-β (Aβ) deposition. Mice expressing CD33M have increased Aβ levels, more diffuse plaques, fewer disease-associated microglia, and more dystrophic neurites compared to 5XFAD control mice. Conversely, CD33m promotes plaque compaction and microglia-plaque contacts, and minimizes neuritic plaque pathology, highlighting an AD protective role for this isoform. Protective phenotypes driven by CD33m are detected at an earlier timepoint compared to the more aggressive pathology in CD33M mice that appears at a later timepoint, suggesting that CD33m has a more prominent impact on microglia cell function at earlier stages of disease progression. In addition to divergent roles in modulating phagocytosis, scRNAseq and proteomics analyses demonstrate that CD33m^+^ microglia upregulate nestin, an intermediate filament involved in cell migration, at plaque contact sites. Overall, our work provides new functional insights into how CD33, as a top genetic susceptibility factor for AD, modulates microglial cell function.

**Graphical Abstract:**

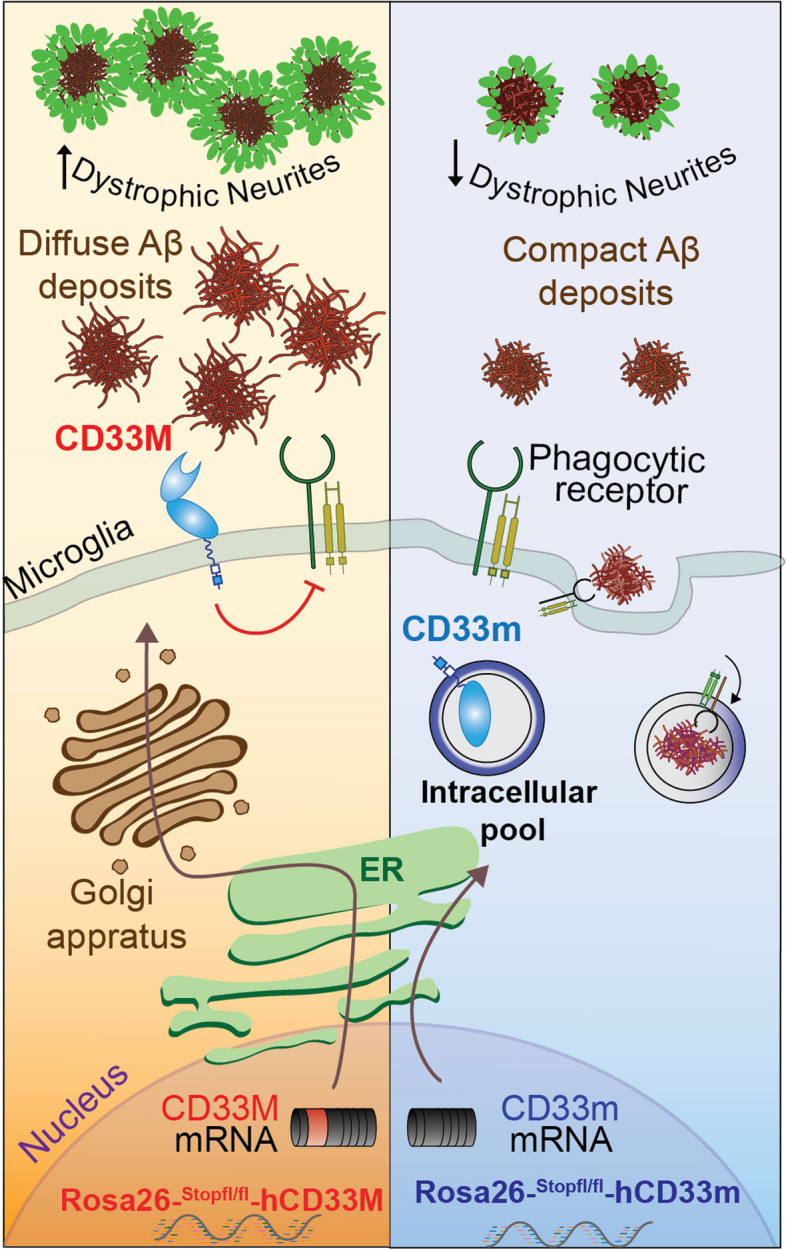

**Supplementary Information:**

The online version contains supplementary material available at 10.1186/s13024-024-00734-8.

## Background

Late onset Alzheimer’s disease (LOAD) arises from a complex interplay between genetic and environmental factors. Genome-wide association studies (GWAS) suggest that approximately 70% of Alzheimer’s disease (AD) risk is attributable to genetic factors [1, 2]. Elucidating the functional aspects of identified genetic factors can facilitate our understanding of the underlying mechanisms contributing to disease pathogenesis. Human genetics, along with mouse models, cumulatively point to immune cells in the brain, called microglia, as being critical in AD pathogenesis through numerous functions, including their ability to phagocytose amyloid beta (Aβ) [3–7]. Of the numerous genes identified by GWAS that are linked to AD risk, a single nucleotide polymorphism (SNP) in the *CD33* gene is one of the top ranked susceptibility loci [8, 9]. In fact, among the top ten AD risk genes conferring small effects, *CD33* was the only one that showed significant effects in combination with *APOE ε4* allele, suggesting that the impact of CD33 on AD susceptibility can be more profound when combined with other major risk factors [10]. CD33 is a member of the immunomodulatory sialic acid-binding immunoglobulin-type lectin (Siglec) family that is expressed on myeloid cells. In the brain, CD33 is predominantly expressed by microglia and its immunoregulatory roles are of great interest in the context of AD pathogenesis [11, 12].

The common AD-associated SNP in *CD33,* rs3865444, is located in the promoter region and is believed to be a functional proxy of a nearby SNP, rs12459419, located four nucleotides into exon-2. This latter SNP modulates mRNA splicing [11, 12]. The common *CD33* allele (rs12459419C) favors the production of a long protein isoform known as human CD33M (CD33M; M=major) and is associated with increased susceptibility to AD relative to a minor *CD33* allele (rs12459419T) [13]. The minor AD protective *CD33* SNP enhances exon-2 skipping, which results in enhanced production of a short protein isoform known as CD33m (m = minor) that lacks the glycan binding IgV domain [8]. While the common allele (rs12459419C) results in generation of approximately 90% CD33M and 10% CD33m, the protective allele (rs12459419T) shifts this ratio to 70% for CD33M and 30% for CD33 isoform [11]. CD33m was originally thought to deliver its protective effects through a loss-of-function stemming from decreased expression of CD33M, however, several recent studies implicated a gain-of-function role for CD33m that may contribute to decreased AD risk. First, another variant of *CD33* (rs201074739), which is a null allele, is not AD protective [14]. Second, our group and others independently provided evidence supporting a gain-of-function role for CD33m in microglia in vitro, with the most striking phenotype being an enhancement in phagocytosis [15, 16]. It is noteworthy to mention that the protective *CD33* allele appears to be derived in humans and is absent in mice and non-human primates [17]. In this regard, an important consideration in studying the impact of CD33 in AD is the lack of functional conservation between mouse CD33 (mCD33) and human CD33 (hCD33) [18]. CD33 from mice and humans have several fundamental differences: (i) mCD33 lacks an immunoreceptor tyrosine-based inhibitory motif; (ii) mCD33 pairs with Dap12; (iii) mCD33 and hCD33 have different ligands; and (iv) the short isoform (CD33m) is not generated in mice. Accordingly, these differences call for the need of human-specific systems to study CD33 effects in the context of AD.

Directly testing a gain-of-function role for CD33m can be challenging as human cells express both CD33 isoforms, making it difficult to deconvolute isoform-specific phenotypes. To tackle these challenges, and to enable the study of individual hCD33 isoforms in vivo, we previously generated and reported two transgenic (Tg) mouse models that express either human CD33M or CD33m in the microglial cell lineage and demonstrated that the two hCD33 isoforms have opposing roles in regulating phagocytosis in microglia in vitro [15, 19]. Moreover, we provided evidence towards the gain-of-function effects of CD33m at functional (enhanced phagocytosis) and transcriptional levels (scRNAseq) [15]. Although the phenotypic aspects of hCD33 isoforms, such as their impact on phagocytosis have been partially explored in vitro [15, 16, 19], the impact of each isoform on microglial cell function in vivo - particularly in the context of AD pathogenesis - has not yet been investigated.

Herein, motivated to deconvolute the function of the two AD-related protein isoforms of hCD33 in vivo, we crossed CD33M and CD33m transgenic mice with the 5XFAD model of amyloidosis [20]. Immunofluorescence microscopy (IF), transcriptomics, proteomics, and biochemical analyses were used to examine the impact of each isoform on microglial cell response and Aβ-induced pathogenesis. We show that hCD33 isoforms differentially modulate microglia and have opposing effects on Aβ accumulation, and plaque composition. CD33M-expressing mice have more diffuse Aβ plaques while CD33m-expressing mice have a more compact plaque phenotype and show increased number of plaque-associated microglia. Accordingly, the gain-of-function role for CD33m goes beyond decreasing the amount of toxic Aβ aggregates, to increasing the number of Aβ deposits with inert dense cores, which strongly correlates with decreased number of dystrophic neurites. Overall, this study is a major step forward towards elucidating the in vivo roles of human-specific CD33 protein isoforms in microglia, further deciphering the functional aspects of this GWAS-identified risk factor, and helping to define early events that contribute to AD pathogenesis.

## Methods

### Animals

The Rosa26-Stop^fl/fl^-hCD33M/m mice were generated on a C57BL/6 genetic background and described previously [15, 19, 21]. The CX3CR1^Cre^ (B6J.B6N(Cg)-Cx3cr1^tm1.1(cre)Jung/J^) and 5XFAD (B6.Cg-Tg(APPSwFlLon,PSEN1*M146L*L286V) 6799Vas/Mmjax) mice were obtained from the Jackson Laboratory. All hCD33 transgenic mice used in this study were homozygous for mCD33. To generate 5XFAD mice expressing either hCD33M (Cx3cr1^Cre+/+^-hCD33M^+/−^) or hCD33m (Cx3cr1^Cre+/+^-hCD33m^+/−^), we crossed (Cx3cr1^Cre+/+^-hCD33M^−/+^ or Cx3cr1^Cre+/+^-hCD33m^−/+^ mice with 5XFAD^−/+^ mice and confirmed the progeny containing hCD33 and 5XFAD with designated primers [15, 19, 21]. Breeders containing either of the hCD33 transgenes were homozygous for CX3CR1^Cre^ so that all the experimental mice contained a single copy of CX3CR1^Cre^. The 5XFAD control mice used in our study were obtained from either of the CD33M or CD33m breeders. Table 1 provides a summary of the genotype of all mice used in this study.

**Table 1:** Genotype of all the mice used in this study

**Group of mice**	CX3CR-Cre	CD33M	CD33m	5XFAD
**5XFAD Control**	*CX3CR1-Cre*^*−/*+^	*CD33M*^*−/−*^	*CD33m*^*−/−*^	*5XFAD*^*−/*+^
**CD33M 5XFAD**	*CX3CR1-Cre*^*−/*+^	*CD33M*^*−/*+^	*CD33m*^*−/−*^	*5XFAD*^*−/*+^
**CD33m 5XFAD**	*CX3CR1-Cre*^*−/*+^	*CD33M*^*−/−*^	*CD33m*^*−/*+^	*5XFAD*^*−/*+^
**Non-5XFAD Control**	*CX3CR1-Cre*^*−/*+^	*CD33M*^*−/−*^	*CD33m*^*−/−*^	*5XFAD*^*−/−*^
**Non-5XFAD CD33m**	*CX3CR1-Cre*^*−/*+^	*CD33M*^*−/−*^	*CD33m*^*−/*+^	*5XFAD*^*−/−*^

All animals were maintained in ventilated racks (Tecniplast, Green Line) and cage environmental enrichment comprising 5 cm diameter plastic tubes and nesting material (“Nestlets”, Ancare Inc.). Animals were fed irradiated chow (LabDiets, 5053) and were housed with a 12 h/12 h light–dark cycle. All protocols were in accordance with the Canadian Council on Animal Care (CCAC) and were approved by the Animal Care and Use Committee at the University of Alberta.

### Immunofluorescence (IF) staining

Half brain sections were fixed in 4% PFA at 4 °C for 24 h, followed by incubation with 30% sucrose (4 °C) for a minimum of 72 h. The tissue was then embedded in embedding medium for frozen tissue specimens (OCT, Thermo Scientific) and stored at -80 °C until being further processed by cryostat (Thermo Scientific). Coronal Sects. (20 μm) within the hippocampus region were collected and a minimum of seven sections per sample were mounted onto Superfrost Plus microscope slides (Thermo Scientific) and stored at -80 °C.

For IF staining, slides were incubated with PBS containing 5% goat serum and 0.1% Triton X-100 for 15 min at room temperature. For specific antibodies, (nestin and Ki-67), heat-induced antigen retrieval was needed, therefore, slides were removed from -80 °C, allowed to adjust to room temperature for 20 min and washed with PBS prior to heat-induced antigen retrieval. Slides were then incubated in 300 ml of antigen retrieval solution, (citrate, pH 6.0; G-Biosciences), at 85 °C for 20 min. Slides were removed, allowed to cool for several minutes, soaked in room temperature PBS for 15 min, followed by a wash in PBS for 10 min prior to adding blocking solution. Slides were then further treated with blocking solution containing 5% goat serum in PBS-T (PBS containing 0.2% Tween-20), followed by incubation with 500 μl of 5% goat serum containing primary antibodies overnight at 4 °C. The following primary antibodies were used in our experiments: anti-Iba1 (rabbit monoclonal, FUJIFILM Wako Chemicals, 1:600 dilution), anti-Aβ (MOAB-2, abcam, 1:1000), anti-Ki-67 (B56, abcam, 1:100), anti-CD68 (Biolegend, 1:100), anti-mouse Clec7a (Invivogen, clone R1-8g7, 1:200) and anti-LAMP1 (1D4B, abcam, 1:200). The slides were washed three times in PBS-T the following day and incubated with the secondary antibodies (AF568 or AF555-conjugated anti-rabbit, AF488-conjugated anti-rabbit, AF647-conjugated anti-mouse, and AF555-conjugated anti-rat, all used at 1:500 dilution) for 1 h, followed by three more washes in PBS-T. To minimize the fluorescent background, autofluorescence quenching kit (TrueVIEW) was used as per the manufacturer’s protocol. Lastly, the slides were incubated with Hoechst (1:2000 dilution of 10 mg/ml stock solution) for 15 min and cover-slipped with permanent mounting medium (TrueVIEW).

Thioflavin-S (Thio-S) staining of amyloid aggregates was performed as described previously [22]. Briefly, sections were stained with 150 µM Thioflavin-S (Sigma) solution in 40% ethanol for 10 min at room temperature. Slides were then washed with 50% ethanol, followed by two washes in PBS prior to mounting coverslips.

### Microscopy

Fluorescence microscopy was performed with the LSM 700 laser scanning confocal microscope (ZEISS), equipped with Axiocam 702 mono camera (ZEISS) and the images were captured at 10X magnification. A minimum of five brain sections from each animal were assessed for analysis. Confocal microscopy images were captured with the same microscope in confocal mode (software: Zen2.6 Black edition, ZEISS) and at 63X magnification oil immersion objective (N.A. 1.4) at 1024 × 1024 pixel resolution. The images were collected at a depth mid-way into the specimen, and the brightest focal plane with sharp morphological features was selected using the live acquisition feature and a 1 AU pinhole that enables a high-resolution image to be collected. All the quantitative analysis were on area of the 2D surface, and in cases wherein normalization was needed, we used a 2D measurement (e.g. imageframe area in mm^2^). Sample identity was blinded for all analyses and images were processed with Zen2.6 Blue edition software (ZEISS).

### Image analyses and quantification

All the global analyses were performed on widefield images captured from the hemi-brains. This included Aβ plaque burden, number of Aβ deposits, percentage of ThioS^+^ deposits, and Iba1 density in the whole brain as well as region-specific analysis of overall Aβ levels. In this regard, the total area of the detected signal was quantified and normalized to the total area of the brain frame. For the analyses performed on individual plaques, images captured in the confocal were used. The details of analyses performed on plaques are as follows:

#### Aβ and Thioflavin-S area for individual plaques

For measuring total Aβ area within each deposit, the plaque area was adjusted to be within the analysis frame and Aβ or ThioS fluorescent signal was distinguished by thresholding. The total area of Aβ/ThioS within the frame was then measured and recorded per plaque. Classification of Thio-S positive plaques were done by scrolling through the z-stack and judging manually. Plaque compaction was measured through dividing the Thioflavin-S area by the total Aβ area. This was calculated both globally (in high plaque density regions) as well as for individual Aβ deposits.

#### Plaque-associated microglia

The area of each plaque deposit was selected by a frame and the total number of plaque-associated microglia was calculated by counting the total number of Iba1 positive nuclei (stained with Hoechst) within the allocated frame region. To measure plaque-associated microglia density, this number was divided by the area of plaque within the frame region.

#### Microglia-plaque interface

Only for plaques with a ThioS core, the area of each ThioS^+^ plaque was selected by a frame and the thresholding for the ThioS channel was adjusted in a way that only the perimeter of the core would be quantified. The Iba1 signal overlapping with ThioS within the perimeter was then measured by the software and the overlap was quantified as a percentage of total perimeter.

#### Quantification of CD68 per Field of view

Images captured at 63 × magnification from the subiculum or cortex were used as Field of view (FOV). The CD68 area inside Iba1 was measured and quantified by the software. A total of 8 FOV, were quantified for each group (*n* = 5/genotype).

#### Percentage of internalized Aβ by microglia

Microglia interacting with Aβ deposits were selected and a barrier mask for the cells was defined based on the Iba1 signal. The total area of Aβ within the mask was measured, quantified, and normalized to the total area of Iba1.

#### Quantification of Ki-67^+^ microglia

A total 20 confocal images of subiculum and cortex (20X magnification) were collected and the number of total Ki-67^+^ microglia in each FOV was counted and graphed (*n* = 5 mice per genotype).

#### Quantification of Clec7a per FOV

Images captured at 63X magnification from the subiculum were used as FOV. The total area of Clec7a signal overlapping with Iba1 was measured and normalized to the total Iba1 area and the percentage of overlap was analyzed by the software. A total of of 8 FOV, were quantified for each group (*n* = 5/genotype).

#### Quantification of nestin levels in plaque-associated microglia

For measuring total nestin area per plaque, the plaque area was adjusted to be within the analysis frame (FOV). Nestin fluorescent signal was distinguished by thresholding and the area of nestin within the frame was quantified per FOV. The analysis frame (FOV) was of constant size for all images. Approximately 30 plaques from 3 mice were quantified per genotype.

#### Quantification of LAMP1^+^ dystrophic neurites (DNs) area in dorsal subiculum

The total area of LAMP1^+^ spheroids within the dorsal subiculum and parts of the frontal cortex adjacent to the subiculum were analyzed and quantified as the percentage of the total brain frame.

#### Quantification of DN area (μm^2^) in individual neuritic plaques

The total area of spheroid within each neuritic plaque was measured and recorded. A total of 400 plaques from 10 mice per cohort were used for this analysis.

### Extraction of soluble and insoluble Aβ from mouse brain

Half brain samples were individually homogenized in sterile PBS containing protease (cOmplete, Roche) and phosphatase inhibitor cocktails (Thermo Fisher Scientific). Homogenization was done using ceramic magnetic beads (2.8-mm ceramic beads; Bertin Technologies SAS) in an Omni bead Ruptor system (3.2 M/s shake speed, 10 s rupture, 10 s break, three repeats) to obtain 10% (w/v) homogenates.

Extraction of soluble and insoluble Aβ was done as described previously with slight modifications [23]. Briefly, 130 µL of homogenate was thawed on ice and mixed with an equal amount of 2% Triton X-100, to achieve a final concentration of 1% in the homogenate. Samples were then incubated on ice for 15 min, while being vortexed every 5 min, followed by ultracentrifugation at 100,000 rcf (4 °C for 15 min). The supernatant was extracted and tested for total protein concentration with BCA assay. The pellet (insoluble fraction) was either resuspended in PBS and sonicated for 1 min for proteinase K (PK) treatment and ApoE quantification, or monomerized by addition of formic acid (final concentration of 70% (v/v) for Meso Scale Discovery assay. The volume of PBS used for resuspension of the pellet was adjusted based on the protein concentration of the soluble fraction.

For PK digestion, the insoluble fraction was aliquoted and treated with PK for 1 h at 37 ºC. The remaining material were then monomerized by addition of formic acid at a final concentration of 70% (v/v) and sonicated for 1 min. The solutions were neutralized (1:20) with neutralization buffer (1 M of Tris base, 0.5 M of Na_2_HPO4, 0.05% NaN_3_ (w/v)) prior to measurements. Aβ_1-42_, Aβ_1-38,_ Aβ_1-40_ levels were quantified by an electrochemiluminescence-linked immunoassay (Meso Scale Discovery (MSD), Assay 2) as per manufacturer’s protocol. Human Aβ oligomer levels in the soluble fraction were quantified using a solid phase sandwich ELISA kit (IBL America). ApoE levels were quantified in both the soluble and insoluble fractions using a Mouse Apolipoprotein E (APOE) ELISA Kit (Mybiosource). The plates were read on a M5 SpectraMax fluorescence plate reader (Molecular Devices) and on the SECTOR Imager 6000 and data analysis was performed using the MSD DISCOVERY WORKBENCH software v.2.0.

### Proteomics

#### Tissue homogenization and sample preparation

For whole proteome analysis of each mouse genotype, individual half brains (*n* = 5 male mice per group) were homogenized using a glass homogenizer in urea-based lysis buffer (8 M urea, 100 mM Tris pH 8.5, 1% SDS, 5 mM EDTA, 1 mM AEBSF, 1 mM PMSF and 4 mM IAM) to obtain 10% w/v homogenates. A round of probe sonication (30% amp, 2 s on/ 2 s off, 2 min) was applied, followed by incubation in the dark on ice for 15 min. Lysates were clarified by centrifugation and total protein concentration of each sample was determined in the recovered supernatant by the BCA assay.

A total of 50 µg of protein from each brain lysate were processed using ProTrap XG cartridges [24] (Proteoform Scientific inc.) following the manufacturer’s protocol with some modifications. Briefly, 100 mM NaCl was added into each sample and the volume was adjusted to 100 µL. Proteins were precipitated in acetone (1:4 ratio) directly on the filtration cartridge at R.T. for 30 min. The cartridge was spun down at 2500 rcf for 2 min, and the protein pellet was washed once with acetone. The pellet was resuspended in 100 µL of 8 M urea by vortexing for 30 s, bath sonication for 10 min, and incubation at R.T. for 30 min. The urea in the samples was diluted by addition of 400 µL of 100 mM Tris buffer (pH 8). Proteins were reduced (10 mM DTT) and alkylated (25 mM iodoacetamide) at 37 °C for 30 min, then 25 mM DTT was added. Digestion was initiated by addition of trypsin at a 50:1 (protein:enzyme) mass ratio. Samples were incubated at R.T. overnight. The reaction was then quenched by addition of trifluoroacetic acid (TFA) (final 2.5%). Peptides were desalted using a SPE column. The cartridge was primed (300 µL ACN), equilibrated (300 µL of 0.1% TFA in water), loaded twice, and washed (300 µL 5% ACN, 0.1% TFA in water). Peptides were eluted (300 µL of 50% ACN, 0.1% TFA in water) into a new tube and dried down using a speedvac and stored at -20 °C until LC–MS/MS analysis.

#### Mass spectrometry and data analysis

Samples were analyzed using a nanoflow-HPLC (Thermo Scientific EASY-nLC 1200 System) coupled to an Orbitrap Fusion Lumos Tribrid Mass Spectrometer (Thermo Fisher Scientific inc.) in data independent acquisition mode. Digested peptides were recovered in buffer A (3.9% ACN, 0.1% formic acid in water). Reverse phase separation of the peptides was done with an Aurora Ultimate™ analytical column (25 cm × 75 µm ID with 1.7 µm media, IonOpticks). Peptides were eluted with a solvent B gradient (0.1% FA in 80% ACN) for 120 min. The gradient was run at 400 nL/min with analytical column temperature set at 45 °C. DIA analysis was done as reported by Mehta et al. 2022 with some modifications [25]. Full scan MS^1^ spectra (350—1400 m/z) were acquired with a resolution of 120,000 at 200 m/z with a normalized AGC Target of 200% and a maximum injection time of 20 ms. MS^2^ was acquired in the linear ion trap, ACG target value for fragment spectra was set to 2000%. Twenty-eight 38.5 m/z windows were used with an overlap of 1 m/z. Resolution was set to 30,000 using a dynamic maximum injection time and a minimum number of desired points across each peak set to 6.

DIA data analysis was performed in the software Spectronaut (v17) using direct DIA analysis workflow using default settings. The database for the searches was the Uniprot mouse proteome (2021, 55,336 sequences) with the hAPP and hCD33 sequence added for the Tg lines. Trypsin/P was selected as the digestion enzyme with a maximum of two missed tryptic cleavages and the search was performed with a maximum false discovery rate of 1% for peptides. Carbamidomethylation (C) was added as fixed modification and, deamidation at N/Q, and M oxidation were set as variable modifications. Before pairwise comparison of the populations under study, protein abundance variability was corrected by normalizing abundance using a global approach on the abundance average. Ratios for the comparisons between all 5XFAD groups—CD33M 5XFAD versus 5XFAD control, CD33m 5XFAD versus 5XFAD control, CD33M 5XFAD versus CD33m 5XFAD, as well as the non-5XFAD control versus non- 5XFAD CD33m group—were generated and an unpaired t-test was applied for differential abundance testing, and the list of candidates was generated for significant proteins with *p*-value ≤ 0.05 and fold change ≥ 1.5 for each comparison. GO analysis for the selected candidates was performed directly in Spectronaut, over and underrepresented GO entries with *p*-value ≤ 0.05 were considered significant. Volcano plots between paired groups (CD33M 5XFAD versus 5XFAD control, CD33m 5XFAD versus 5XFAD control, CD33M 5XFAD versus CD33m 5XFAD and non-5XFAD control versus non-5XFAD CD33m, were generated on Prism (v9) using the list of proteins on each individual comparison. The triplot was generated using excel by initially graphing the log_2_(ratio) of each comparison on each side of a triangle. The intersection of lines between the specific fold change value and the vertex at the opposite end of the triangle corresponds to the proteomic correlation between the populations compared. The location of each circle on the graph indicates the association of that protein to the specific comparison. Identifications closer to the vertex, correspond to proteins with higher association to that genotype, while identifications closer to the center of the triangle indicate similar levels of that protein in all comparisons. From the lists of candidates generated, proteins with increased abundance to the CD33m and CD33M populations were extracted and highlighted in the triplot (blue and red, respectively). Additionally, proteins with GO annotations associated with brain processes were extracted from the list of identified proteins [26], and cell-specific markers were defined based on the single cell information from *proteinatlas.org*. A heatmap of the normalized abundance (Z-score) was built using “superheat” R package.

### Isolation of adult mouse microglia

Isolation of microglia was performed as described previously [27]. Briefly, mice were perfused with 15 mL of ice-cold HBSS buffer containing Actinomycin D (5 μg/mL) and Triptolide (10 μM). Brain was then extracted and stored in 10 mL storage buffer containing Actinomycin D (5 μg/mL), Anisomycin (27.1 μg/mL) and Triptolide (10 μM) at 4 °C. Minced brains were homogenized in ice cold storage buffer with a 5 ml syringe plunger through a 40 μm filter (Corning) under sterile conditions. The cell suspension was then transferred to a 15 mL tube and centrifuged for 5 min at 4 °C (300 rcf). After centrifugation, the pellet was collected and resuspended in 10 mL of ice cold 40% Percoll (Sigma) diluted in 1x (final) HBSS and centrifuged (30 min, 500 rcf, 4 °C). After carefully removing the Percoll layer containing myelin debris, microglia were pelleted. After centrifuging (5 min, 300 rcf, 4 °C), the collected cell pellet was washed with 10 mL of ice-cold 1 × HBSS buffer. Samples were resuspended in 50 μl of ice-cold flow buffer (0.5% BSA, 1 mM EDTA, in 1 × PBS, Sterile Filtered) containing antibodies targeting Cd11b (BV510, clone ICRF44, Biolegend), CD45 (APC/Cy7, clone 30-F11, Biolegend), and Cx3cr1 (PerCP/Cy5.5, clone SA011F11, Biolegend) from Biolegend all at 1:200 dilution for 20 min on ice. Following incubation, the samples were washed with ice cold flow buffer, centrifuged (5 min, 300 rcf, 4 °C), and resuspended in 700 μl of ice-cold cell sorting buffer (1 × HBSS containing 10% FBS and 1 mM EDTA) in preparation for cell sorting. An estimated 70,000 microglia (CD11b^+^, CD45^+^, Cx3cr1^+^) were sorted using the 100 μm nozzle at a sorting speed of approximately 3500 events/sec. We collected sorted samples in 1.7 ml Eppendorf tubes. Sorted cells were centrifuged (300 rcf, 5 min, 4 °C) and the supernatant was removed. Pelleted cells were resuspended in 100 μL PBS + 0.1% BSA and 10μL of the sample immediately counted using a Neubauer chamber using 0.4% Trypan blue solution (Thermo Fisher). For experiments, samples with a viability of over 95% were used. Cells were resuspended in PBS in an appropriate volume to achieve a concentration of 1000 cells/mL.

### Single-cell RNA sequencing

Cell suspension was used to generate the gel-beads + cell emulsion by the 10X Chromium Controller (PN-1000202) using the Chromium Next GEM Single Cell 3′ GEM, Library & Gel Bead Kit v3.1 (PN-1000121), Chromium Next GEM Chip G Single Cell Kit (PN-1000120) and Single Index Kit T Set A, (PN-1000213). Reverse transcription, cDNA amplification, library preparation, and sample barcoding were performed following the available manufacturer’s protocol. Finally, sample libraries were pooled and sequenced in Illumina HiSeq P150 (Sequencing type: Paired-end, single indexing) to an average depth of ~ 50,000 reads per cell. For our scRNAseq studies, 5XFAD, CD33M^+^ 5XFAD and CD33m^+^ 5XFAD were all run from the same cohort of mice, while the non-5XFAD mice were from a separate cohort that was run at a different date under identical conditions.

#### Library preparation

We carried out scRNAseq analysis on microglia extracted from the whole brain of two pooled 5XFAD mice from all three genotypes at eight months, along with one age-matched control non-5XFAD. The FACS-isolated cells were processed on the 10 × chromium controller following the 10 × Genomics Next GEM Single Cell 3’ GEM, library, and v3.1 Gel Bead kit (10 × Genomics; Cat. No. 1000121) and sequenced the samples on the Illumina HiSeq P150 sequencer at Novogene corporation inc. The samples were paired-end, single index sequenced at an average read depth of 50,000 reads per cell. The resulting BCL files were demultiplexed into FASTQ files and aligned to a custom *Mus Musculus* 10 (MM10) reference genome, adjusted to include *Clec7a*, a polymorphic pseudogene. The *Clec7a* annotation was added to the 10 × Genomics pre-built MM10 genome (2020-A) GTF file from the 10 × Genomics parent GTF file (gencode.vM23.primary_assembly.annotation.gtf.gz). The final custom genome was created by combining the MM10 genome (2020-A) FASTA file with the *Clec7a* modified GTF file using the cellranger mkref function in the 10 × cell ranger pipeline (v3.0.0). Finally, the samples were aligned the custom genome using the cellranger count function to generate barcoded and sparse matrices, both raw and filtered, along with BAM files for downstream analyses.

#### Quality control, dimensionality reduction and clustering

Quality control, dimensionality reduction and initial clustering was performed in the R statistical environment (v4.1.2) using Seurat (v4.0; https://github.com/satijalab/seurat). A Seurat object was created per dataset to include genes expressed in a minimum of 3 cells and cells expressing a minimum of 200 genes using the CreateSeuratObject() function. The object was further refined to remove doublets and multiplets by removing cells with high gene counts (> 3000 genes) and dead cell by removing cells high percentages of mitochondrial genes (> 10%). All datasets were merged using the merge() function and normalized using the SCTransform() function according to the binomial regression model (Highly variable features = 3000, nCount and mitochondrial genes regressed).

Dimensionality reduction was performed using RunPCA(), FindNeighbors() (Dimensions = 15) and FindClusters() functions. 25 PCs were used for downstream analyses as determined by the PCA elbow plot. The FindClusters() function was run at multiple resolutions, ranging from 0 to 1, separated by 0.1. All clustering resolutions (0 to 1) were plotted on a tree generated by the Clustree package using the clustree() function. The 0.5 resolution was chosen for clustering based on the most stable level as identified by clustree().

The final clustering of the dataset was performed in a Jupyter notebook (v6.0.3) running a python environment (Python 3.8.3) and using Single Cell Clustering Assessment Framework (SCCAF) package (v0.0.10, https://github.com/SCCAF/sccaf). The SeuratObject was converted to an h5ad file using SeuratDisk by first converted the RDS file to an h5Seurat file using the Saveh5Seurat() and converting the h5Seurat file to an h5ad file using the convert() function (Destination = h5ad). The h5ad file was read into the python environment using Scanpy (v1.6.0)[28]. The clustering was refined using the SCCAF_optimize_all() function (minimum accuracy = 95%, iterations = 150). The machine learning algorithm iteratively clustered the dataset until a 95% self-projection accuracy was reached. The final clustering iteration was projected onto a UMAP using the sc.pl.umap() function.

#### Batch effect correction

We used Harmony (v0.1.0), Seurat Integration and FastMNN to correct for possible batch effects.

#### Harmony

Quality control and normalization was performed using Seurat (v5.0) as described above. Dimensionality reduction was performed using RunPCA() and RunHarmony() was used to correct for batch effects. The corrected embeddings were used to cluster the data as described above, specifying ‘reduction = harmony’ in the FindNeighbors() and FindClusters() functions.

#### Seurat Integration

Quality control and normalization was performed using Seurat (v5.0) as described above. Dimensionality reduction was performed using RunPCA() and RunUMAP. The layers in the original clusters were integrated using IntegrateLayers() (method = CCAintegration) and the integrated embeddings were used to cluster the data as described above, specifying ‘reduction = integrated.dr’ in the FindNeighbors() and FindClusters() functions.

#### FastMNN

Quality control and normalization was performed using Seurat (v5.0) as described above. Dimensionality reduction was performed using RunPCA() and RunFastMNN() was used to correct for batch effects. The corrected embeddings were used to cluster the data as described above, specifying ‘reduction = mnn’ in the FindNeighbors() and FindClusters() functions.

All datasets were converted to an h5ad file by first converting the Seurat V5 object into a V3 object by converting the SCT from Assay5 to Assay. SeuratDisk was used to convert the new V3 RDS file to h5ad and the data was re-clustered using SCCAF as described above.

#### Pseudobulk comparisons

All pseudobulk analyses were performed on the merged Seurat object, creating pseudobulk metadata using the AverageExpression() function of the library idents and determining the differentially expressed genes between pairs of libraries using the FindMarkers() function. Finally the Volcano plots were plotted through EnhancedVolcano (v.1.20.0) using the EnhancedVolcano() function.

#### Functional gene ontology pathway analysis

Functional Gene Ontology pathways were identified through the gProfiler [29] web server (https://biit.cs.ut.ee/gprofiler/gost) by determining the intersection of differentially expressed genes between libraries, identified using FindAllMarkers() function (only.pos = FALSE, min.pct = 0.25, min.threshold = 0.25), and genes present in the gene ontology (molecular function, cellular component and biological processes), KEGG and REACTOME databases. Pathways were plotted on an alluvial plot through the ggalluvial package (v0.12.3) using the ggplot() function, retaining only pathways with a *p*-value < 0.5.

#### *hCD33* transgene comparison

The *hCD33* gene sequence The *hCD33* gene sequence (CD33 molecule [Homo sapiens] Gene ID:945) was added to Mus Musculus 10 genome using the cellranger mkref function as described above and the custom reference genome was used to determine the levels of *hCD33*.

### Behavioral assays

Mice were habituated in the behavior suite for one hour prior to each experiment. White noise was played via a sound device during habituation and during each experiment. Mice were subject to the open-field assay, followed by the light–dark box, and the spontaneous Y-maze. Each test was conducted on different days.

#### Light − Dark box

The light–dark box was divided into two sections—an open, light-exposed Sect. (25 cm × 40 cm) enclosed by clear acrylic walls, and a dark Sect. (17.5 cm × 40 cm) enclosed by black acrylic walls with a black plastic cover preventing light exposure from above the arena. The arena features a 5-cm width opening between the open and enclosed sections. Mice were placed in the light-exposed section and were able to freely navigate between the light and dark regions of the arena for 10 min. Each mouse’s behavior and movement in the 10-min period were recorded and tracked via EthoVision 17 (Noldus, Wageningen, the Netherlands). Tracking was dependent on the mouse’s center-point as detected by EthoVision 17. The amount of time the mouse spent in the light section of the arena during the testing period was tracked by the software.

#### Spontaneous Y-Maze

Mice were placed in the center of a white acrylic Y-shaped maze with 3 equal-length (35 cm × 5 cm) arms. Mice were allowed to freely navigate in the arena for 8 min. Each mouse’s behavior and movement were recorded and tracked via EthoVision 17 (Noldus, Wageningen, the Netherlands). Tracking was dependent on the mouse’s center-point as detected by EthoVision 17. The alternations a mouse made, defined as the number of times a mouse visited each arm sequentially without revisiting a previous arm as determined by the software (ex. moving from arm 1 to arm 2 to arm 3 is counted as an alternation, whereas moving from arm 1 to arm 2 and back to arm 1 is not counted as an alternation). The alternation index is calculated by dividing the number of alternations by the maximum possible alternations as calculated by EthoVision 17.

#### Open field test

Two white cube-shaped (40 cm × 40 cm) arenas, placed side-by-side were used for this experiment, enabling recording of two mice at a time. One mouse was placed in each arena for testing. Each mouse’s behavior and movement in a 15-min period were recorded and tracked via EthoVision 17 (Noldus, Wageningen, the Netherlands). Tracking was dependent on the mouse’s center-point as detected by EthoVision 17, with the nose- and tail-points being defined. The center zone was defined as a 30 cm × 30 cm area in the middle of the arena. After testing, the mouse was placed back into its home cage, and the arenas were thoroughly cleaned with 70% ethanol prior to testing the next mouse.

### Statistical analyses

Data represented as mean ± SD. The D'Agostino-Pearson normality test was used to test for Gaussian distribution of datasets. Differences between the groups were evaluated with one-way Anova followed by the Holm–Sidak Test. A probability of *P* < 0.05 was considered indicative of significant differences between groups.

## Results

### Human CD33 isoforms distinctively alter Aβ plaque burden

**Fig. 1:**
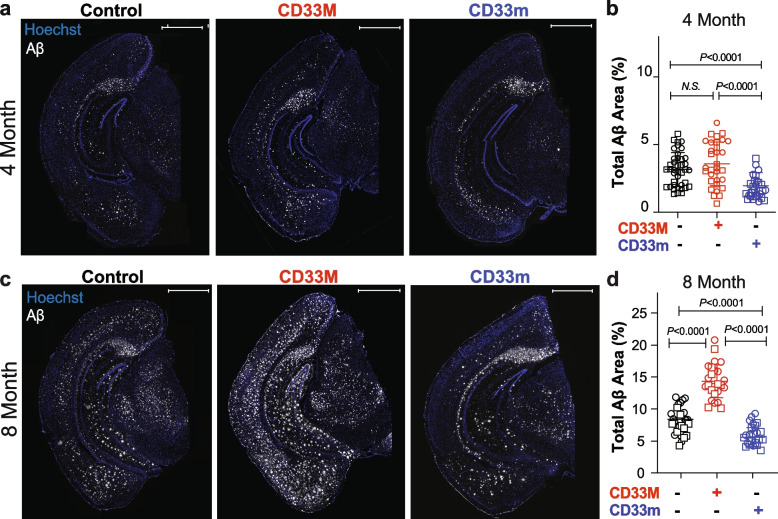
hCD33 isoforms have opposing effects on Aβ accumulation in 5XFAD mice. **a** Representative epifluorescent images of Aβ deposition in 5XFAD mice at 4 months by IF imaging with the anti-Aβ antibody (clone MOAB2, white) and Hoechst (blue). **b** Quantification of total Aβ levels at 4 months in pooled male (squares; *n* = 20, 18, and 12 for control, CD33M, and CD33m, respectively) and female (circles; *n* = 20, 15, and 15 for control, CD33M, and CD33m, respectively) mice. **c** Representative epifluorescent images of Aβ deposition in 5XFAD mice at 8 months. **d** Quantification of total Aβ levels at 8 months in pooled male (squares; *n* = 19, 11, and 10 for control, CD33M, and CD33m, respectively) and female (circles; *n* = 22, 14, and 13 for control, CD33M, and CD33m, respectively) mice. Scale bar = 1000 µm

To study the impact of hCD33 isoforms on the accumulation of Aβ in vivo, our previously developed STOP^flx/flx^-CD33M [19] and STOP^flx/flx^-CD33m [15] Tg mice were crossed with 5XFAD mice [20]. We previously established that these models give physiologically-relevant expression of hCD33 isoforms specifically in microglia when crossed with CX3CR1^Cre^ mice [15]. All experimental mice in this study had a single copy of CX3CR1^Cre^ to drive specific expression of the transgene in the microglia cell lineage and were aged to four or eight months. We confirmed the lack of any statistically significant differences between 5XFAD control mice lacking a human CD33 transgene generated from either CD33M or CD33m mice bred with 5XFAD mice (Suppl. Figure 1). Because of the known sex-dependent differences in accumulated Aβ levels in the 5XFAD model, and to investigate sex-specific impacts from hCD33 isoforms on Aβ deposition, cohorts of both male and female mice were examined (Suppl. Figure 2). Trends were similar in both female and male mice, and we could not detect any sex-specific differences between hCD33 groups compared to the 5XFAD control. However, female mice from all genotypes showed augmented deposited Aβ levels (Suppl. Figure 2a-d), which is consistent with previous reports in 5XFAD mice [20, 30]. We did not observe any significant differences between male and female mice in compaction, total Iba1 density, and plaque-associated microglia density (PAM) (Suppl. Figure 2e-r) [20, 31, 32]. We first conducted immunohistochemistry analysis by fluorescence microscopy of coronal hemi-brain slices stained with an anti-Aβ antibody and quantified the area of deposited Aβ as a percentage of the total brain area (Fig. 1). At four months, there was no significant difference between the 5XFAD control and CD33M group, but CD33m mice had significantly reduced levels of Aβ compared to both 5XFAD control and CD33M mice (Fig. 1a,b). In keeping with previous observations in the 5XFAD model, the dorsal subiculum showed a high Aβ plaque density [20, 33, 34]. At eight months, all cohorts presented significant differences in deposited Aβ levels, with CD33M mice having higher levels of Aβ compared to the other groups, while CD33m mice showed lowest Aβ levels (Fig. 1c,d). Due to the absence of any notable sex driven effects from either of hCD33 isoforms on other variables, we pursued the rest of the analyses in combined sexes.

Regarding Aβ levels, we analyzed two additional parameters: region-specific analysis of deposited Aβ levels in cortex and hippocampus, and the number of Aβ deposits in whole brain. At four months of age, CD33m mice showed less Aβ deposition in the cortex compared to 5XFAD control and CD33M mice, but no differences between the groups were observed in the hippocampus at this timepoint (Suppl. Figure 3a,b). At eight months, Aβ deposition was significantly different between all three groups in the cortex, while in the hippocampus the only significant difference was between CD33M versus 5XFAD control and CD33m mice (Suppl. Figure 3c,d). Quantitative analysis of the number of Aβ deposits pointed to no significant differences between any of the groups at four months (Suppl. Figure 3e). However, by eight months these differences reached statistical significance with CD33m mice showing overall fewer Aβ deposits and CD33M mice showing more Aβ deposits compared to the 5XFAD control mice (Suppl. Figure 3f).

### Expression of hCD33 isoforms significantly alter Aβ plaque composition

**Fig. 2:**
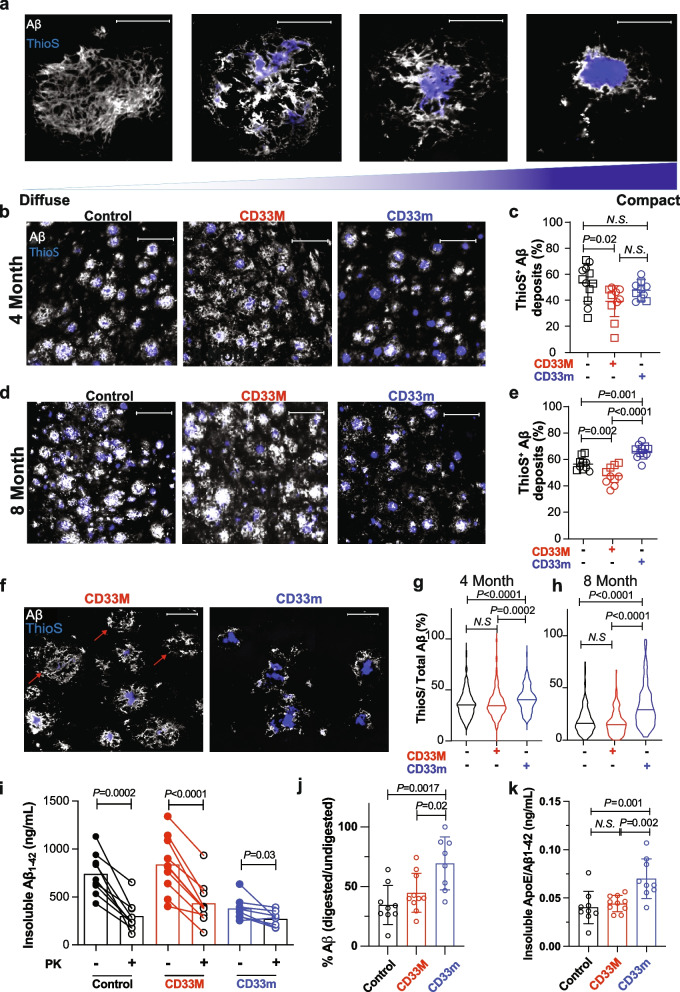
Altered Aβ plaque composition by hCD33 isoforms. **a** Representative confocal fluorescent images showing the different degrees of plaque compaction by co-staining for total Aβ and ThioS. A diffuse plaque without ThioS staining at the far left, a plaque with small ThioS core in the middle, and a highly compact plaque at the far right are presented. Scale bar = 20 µm (**b-e**). Quantification of Aβ deposits containing a ThioS.^+^ core. Representative epifluorescent images of Aβ deposits in the dorsal subiculum of 5XFAD mice at (**b**) 4 and (**d**) 8 months, with co-staining with anti-Aβ antibody (white) and ThioS (blue). Scale bar = 50 µm. Quantification for pooled males (squares; *n* = 5 per genotype) and female (circles; *n* = 5/genotype) mice at (**c**) 4 and (**e**) 8 months. **f** Representative confocal fluorescent images of plaque composition in the dorsal subiculum of 8 months old CD33M and CD33m mice. Scale bar = 20 µm (**g**,**h**) Quantification of plaque composition (ratio of Aβ over ThioS levels) in the dorsal subiculum of 5XFAD mice at (**g**) 4 and (**h**) 8 months. A total of 300 plaques per mouse, from 5 males and 5 females, were quantified for each group. **i** Biochemical characterization of PK-sensitivity of insoluble Aβ_1-42_ from 5XFAD mice. **j** Quantification of the percentage of insoluble Aβ_1-42_ levels after PK digestion compared to not treated with PK. **k** Quantification of the ratio of ApoE to Aβ_1-42_ in the insoluble fraction from 5XFAD mice (*n* = 9, 10, and 9 mice of control, CD33M, and CD33m genotypes, respectively)

Aβ deposits exhibit varying compositions, including dense core and diffuse morphologies (Fig. 2a). Dense core plaques are predominately composed of fibrillar Aβ aggregates and are readily detected with amyloid-binding dyes such as Thioflavin S (ThioS) [35–37]. In contrast, diffuse plaques, typically containing dispersed protofibrils and oligomers, stain weakly with ThioS but can be readily detected with Aβ antibodies. To investigate the impact of hCD33 isoforms on plaque composition, we stained brain slices with ThioS in parallel with an anti-Aβ antibody. Within the whole brain slice, CD33M mice had significantly fewer number of Aβ deposits with a ThioS core at both timepoints (Fig. 2b-e). Unexpectedly, CD33m had a significantly higher percentage of ThioS^+^ deposits compared to the other two groups at eight months Fig. 2b-e). These findings suggest that hCD33 isoforms impact more than just Aβ deposition and have strong effects on plaque composition. Specifically, these observations point to a shift in plaque composition towards a more compact form in the CD33m mice, which is the isoform associated with protective effects in the context of AD [11, 18].

To examine the Aβ plaque composition in greater detail, we carried out quantitative analyses of the dorsal subiculum and cortex at the eight-month timepoint. Consistent with the global analysis of the hemi-brain, we observed the highest deposited Aβ levels in CD33M mice compared to both the 5XFAD control and CD33m mice (Suppl. Figure 4a). ThioS levels in CD33M mice were highest compared to the other two groups, however, CD33m mice also showed higher ThioS levels in the dorsal subiculum compared to the 5XFAD control group (Suppl. Figure 4b). When examining the ratio of ThioS to the amount of total deposited Aβ, the results once again point to CD33m promoting more compact Aβ while CD33M promotes the opposite outcome (Suppl. Figure 4c).

These differences in plaque composition motivated further analysis of individual plaques in dorsal subiculum and cortex (Fig. 2f). At four months, CD33m mice had smaller Aβ deposits than other groups, while CD33M and 5XFAD control showed no differences (Suppl. Figure 4d). However, CD33M had larger ThioS cores (Suppl. Figure 4e). As a result of smaller Aβ deposits, despite no decrease in the size of ThioS core, CD33m promoted higher plaque compaction compared to CD33M and 5XFAD control (Fig. 2g). At eight months, both CD33M and CD33m had a significant impact on the size of Aβ deposits, with CD33M promoting larger Aβ cluster size and CD33m promoting smaller Aβ cluster size (Suppl. Figure 4f). Despite an overall smaller Aβ deposits, CD33m promoted larger ThioS core area in the plaques compared to the 5XFAD control (Suppl. Figure 4 g). Regarding the total number of Aꞵ deposits in the subiculum, CD33M^+^ mice had the highest number of Aꞵ deposits compared to the 5XFAD control and CD33m groups (Suppl. Figure 4 h). There was no significant difference between groups in the number of ThioS^+^ deposits (Suppl. Figure 4i), however, CD33m^+^ mice had the lowest number of diffuse deposits (ThioS^−^) in this region (Suppl. Figure 4j). Quantitative analysis on ThioS core sphericity pointed to significantly highest level of sphericity in CD33m compared to the two other groups while the ThioS core of CD33M mice had lowest level of sphericity (Suppl. Figure 4 k,l). Therefore, CD33m promoted the highest level of plaque compaction compared to other genotypes, while CD33M showed no significant difference compared to the 5XFAD control group (Fig. 2h). It is worth noting that for the analyses on individual deposits, we focused on ThioS^+^ plaques, whereas the global plaque compaction in the dorsal subiculum region did not differentiate between the plaques with or without a ThioS core.

To perform further biochemical analysis on Aβ deposits, we extracted soluble and insoluble Aβ from the snap-frozen half brain of 5XFAD mice at eight months and quantified Aβ_1-42_, Aβ_1-38,_ and Aβ_1-40_ levels by electrochemiluminescence. No significant differences were identified in soluble levels of any of the three Aβ species between the genotypes (Suppl. Figure 5a-c). However, the levels of insoluble Aβ_1-42_ were significantly lower in CD33m mice compared to the other two groups (Suppl. Figure 5d). CD33M mice had significantly higher levels of Aβ_1-38_ and Aβ_1-40_, in the insoluble fraction, compared to CD33m and 5XFAD control (Suppl. Figure 5e,f). As the Aβ_1-42_/Aβ_1-40_ ratio is an indicator of disease progression and cognitive decline [38], we measured this ratio in the insoluble fraction and observed significantly decreased levels of Aβ_1-42_/Aβ_1-40_ in CD33M^+^ mice compared to the other groups (Suppl. Figure 5 g). We also examined the levels of Aβ_1-42_ oligomers in the soluble fraction and although there was a trend towards higher levels in CD33M mice, this difference did not reach significance (Suppl. Figure 5 h). As different forms of Aβ aggregates show different sensitivity to protease digestion [39, 40], we assessed the sensitivity of the Aβ_1-42_ in the insoluble fraction to proteinase K (PK) digestion [41]. A titration of PK was first performed to optimize the concentration that produced the maximum decrease in Aβ levels in the insoluble fraction (Suppl. Figure 5i). Accordingly, a PK concentration of 20 µg/ml was selected and applied to samples from all genotypes, which led to a significant decrease in Aβ_1-42_ levels in all groups (Fig. 2i). The remaining levels of Aβ_1-42_ post-digestion was indistinguishable between all groups, which was likely due to the presence of highly compacted, PK-resistant material. However, the percentage of Aβ_1-42_ relative to the undigested control in each group demonstrated that CD33m mice had significantly less PK-sensitive Aβ_1-42_ compared to the others (Fig. 2j).

As dense, compact plaques have been shown to have higher levels of associated ApoE [42], we quantified total levels of ApoE in the fractionated brain homogenates and observed significantly higher levels of ApoE in the insoluble fraction of CD33m mice in comparison to the 5XFAD control group (Suppl. Figure 6). Normalizing the ApoE levels in the insoluble fraction of each sample to insoluble Aβ_1-42_ levels, CD33m showed the highest levels of ApoE/Aβ_1-42_ than 5XFAD control and CD33M mice (Fig. 2k). Taken together, the combination of or results further supports our earlier observations, suggesting that the protective effects of CD33m can be delivered by a decrease in Aβ levels, which is consistent in biochemical analyses of the insoluble fractions and the total Aβ quantification of half brain sections using IF microscopy (Fig. 1).

### hCD33 isoforms modulate microglial cell response to Aβ deposits

**Fig. 3:**
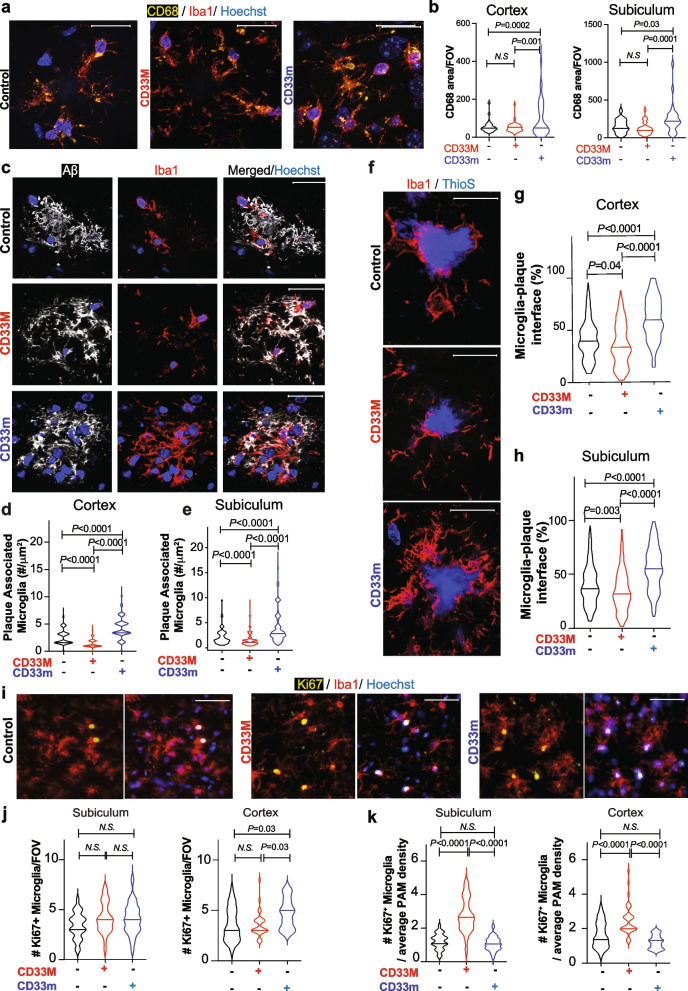
hCD33 isoforms modulate differential response of microglia to Aβ. **a** Representative confocal fluorescent images of CD68 staining of control, CD33M, or CD33m 5XFAD mice at 8 months. IF images are co-stained with anti-Aβ antibody (white), anti-Iba1 (red), and Hoechst (blue). Scale bar = 20 µm. **b** Quantification of total CD68 area measured as the area of CD68 within Iba1 per FOV. A total of 8 FOV, were quantified for each group (*n* = 5/genotype). **c** Representative confocal fluorescent images of plaque associated microglia in control, CD33M, and CD33m mice. IF images are co-stained with anti-Aβ antibody (white), anti-Iba1 (red), and Hoechst (blue). Scale bar = 20 µm. **d**,**e** Quantification of plaque associated microglia normalized to µm^2^ of plaque area at 8 month. A total of 150 plaques in cortex (**d**) and 300 plaques in subiculum (**e**), of 10 mice (5 males and 5 females per group) were analyzed. **f** Representative confocal fluorescent images of microglia-ThioS core interface in control, CD33M, and CD33m mice. IF images are co-stained with anti-Iba1 (red), and ThioS (blue). Scale bar = 20 µm. **g**,**h** Quantification of plaque-microglia interface in 5XFAD mice at 8 months, measured as the percentage area of ThioS perimeter with overlapping Iba1 signal. A total of 150 plaques in cortex (**g**) and 300 plaques in subiculum (**h**), of 10 mice (5 males and 5 females per genotype) were analyzed. **i** Representative confocal fluorescent images of Ki-67 staining of microglia in 8 months-old control, CD33m and CD33m mice. IF images are co-stained with anti-Ki-67 (yellow), anti-Iba1 (red), and Hoechst (blue). Scale bar = 100 µm.** j** Quantification of Ki-67^+^ microglia per FOV measured as the number of macroglia with clear Ki-67 signal in the nuclei. A total of 5 confocal images of subiculum or cortex per mouse were analyzed (*n* = 5 mice/genotype). **k** Number of Ki-67 + microglia per FOV normalized to average PAM density of subiculum or cortex for each genotype

Microglial cell phagocytosis in vitro is increased by CD33m and decreased by CD33M [15, 16, 19, 43]. As a proxy for phagocytosis in vivo, we used co-staining of Iba1 and CD68, as a general marker of reactive phagocytic microglia [44], to quantify the amount of CD68 area within microglial cells in cortex and subiculum (Fig. 3a,b) [45]. At eight months, the CD33m^+^ microglia had significantly higher levels of CD68 area within microglia per FOV compared to 5XFAD control and CD33M microglia (Fig. 3b). Co-staining with anti-Aꞵ antibody, confirmed interactions between internalized Aꞵ with CD68^+^ structures in microglia (Suppl. Figure 7a). We also quantified the internalized Aꞵ levels in PAM and observed decreased levels of internalized Aβ in CD33M microglia, while CD33m microglia had increased levels of internalized Aβ compared to 5XFAD control microglia (Suppl. Figure 7b,c). The decreased and increased levels of internalized Aβ in CD33M and CD33m microglia respectively, could explain the overall trends observed in Aβ levels, plaque compaction, and brain plaque load.

We next analyzed the peri-plaque microglia, also known as PAM, in the subiculum and cortex (Fig. 3c-e). At eight months, CD33M^+^ mice had decreased density of PAM in the cortex and subiculum compared to the other two groups, while CD33m had significantly highest numbers of PAM in both regions (Fig. 3d,e). These differences were also observed at four months in the subiculum (Suppl. Figure 8a). These trends were not due to a global effect on the number of microglia cells, as overall Iba1 levels at eight and four months showed the opposite trend and revealed increased Iba1 density in CD33M mice compared to other groups (Suppl. Figure 9). Of note, the increased Iba1 density could reflect on microgliosis in the CD33M mice or increased inflammatory response due to greater plaque burden.

As microglia promote compaction through biochemical and biophysical mechanisms [45–47], we analyzed the microglia-ThioS core contact sites by quantifying the perimeter of the ThioS core interface that was covered by Iba1 signal in ThioS^+^ deposits in the subiculum and cortex (Fig. 3f-h). We observed a significant increase in microglia-plaque interface in CD33m mice at eight months in both cortex (Fig. 3g) and subiculum (Fig. 3h). These differences were also observed at four months within the subiculum (Suppl. Figure 8b). Accordingly, CD33M mice showed the opposite trend regarding microglia-plaque interface. These results reveal another way that hCD33 isoforms modulate microglia response to Aβ deposits.

Increased numbers of PAM in the CD33m group, despite their lower global Iba1 density, prompted us to investigate the origins of these effects. We started by quantifying the proliferating PAM at eight months through assessing Ki-67 staining, as done by others [48, 49], within microglia in the subiculum and cortex (Fig. 3i). Expression of CD33M or CD33m had no effect on Ki-67^+^ microglia compared to the 5XFAD control in the subiculum. In the cortex, the slightly higher ratio of Ki67^+^ microglia in CD33m^+^ mice suggested they were more proliferative than other groups (Fig. 3j). However, when normalized to the average PAM density, CD33M-expressing microglia had a significant increase in the relative number of Ki-67^+^ PAM compared to other groups (Fig. 3k), which is in line with the higher Iba1 density in these mice (Suppl. Figure 9). These results rule out enhanced proliferation as a crucial mechanism for enhanced numbers of PAM in CD33m mice, suggesting that other mechanisms may be at play.

### A protein implicated in cell migration is upregulated in CD33m^+^ microglia

**Fig. 4:**
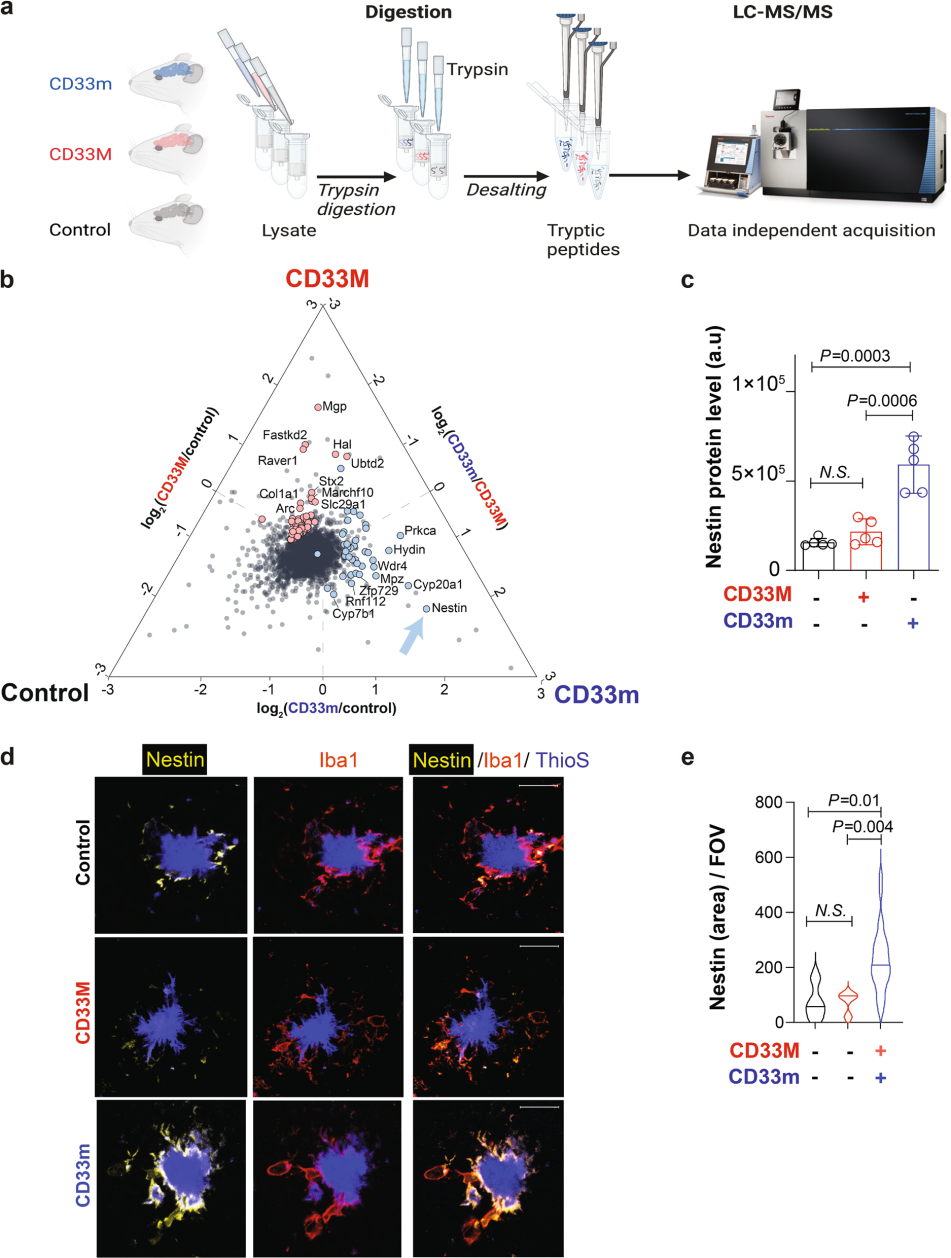
Quantitative proteomics reveal changes in protein abundance in the brain of 5XFAD mice expressing hCD33 isoforms. **a** Schematic representation of the mass spectrometry workflow used for the assessment of global proteome changes in the brain of 5XFAD mice expressing CD33M or CD33m compared to control at 8 months. A total of 5 male mice were used per genotype. On-column digestion and data-independent acquisition mass spectrometry (DIA-MS) was used to quantify protein levels. **b** Correlation of the proteomic changes in the mouse brain for all proteins identified by DIA-MS. The triplot shows protein abundance changes calculated from each pairwise comparison (CD33M vs control, CD33m vs control, and CD33m vs CD33M). Identifications closer to the vertex correspond to proteins with higher association to that genotype, while identifications closer to the center of the triangle indicate similar levels of that protein in all comparisons. Proteins with significant changes (fold change ≥ 1.5 and *p*-value ≤ 0.05) are highlighted. The proteins increased in mouse brains expressing CD33m are colored in blue, while the proteins increased in the CD33M isoform are colored in red. **c** Quantification of nestin in each individual sample as measured by mass spectrometry. **d** Representative confocal fluorescent images of nestin staining of PAM in 5XFAD mice at 8 months in control, CD33m, and CD33m mice. IF images are co-stained with anti-nestin (yellow), anti-Iba1 (red), and ThioS (blue). Scale bar = 20 µm. **e** Quantification of nestin levels in PAM of control, CD33M and CD33m mice. Approximately 30 plaques from 3 mice were quantified per genotype

We next took an unbiased approach to identify proteins that may contribute to the enhanced response of microglia to Aβ plaques in CD33m mice. Accordingly, we applied label-free mass spectrometry to perform global quantitative proteomics on brain homogenates of mice at eight months (Fig. 4a). More than 6000 proteins were consistently identified in each biological replicate by our data-independent acquisition mass spectrometry approach (DIA-MS). On average, 6697 proteins were found in the CD33M mice, 6727 for the CD33m mice, and 6732 for the 5XFAD control. The abundance of all those proteins was quantified in all samples and reported here (Suppl. File 1). Examining differentially expressed proteins revealed numerous differences in global proteome of CD33M or CD33m-expressing microglia compared to the 5XFAD control group (Suppl. Figure 10a-c). Significantly increased and decreased proteins were defined as those with a fold change larger than 1.5 or smaller than 0.66 respectively, and a *p-*value ≤ 0.05. Comparison of CD33M versus 5XFAD control pointed to a total of 47 proteins significantly increased in the CD33M brain, while 96 proteins were decreased. To further address the proteomic changes amongst different genotypes, we repeated the same workflow on non-5XFAD control (Cx3cr1^Cre−/+^hCD33m^−/−^5XFAD^−^) and non-5XFAD CD33m^+^ (Cx3cr1^Cre−/+^hCD33m^+/−^5XFAD^−^) mice (n = 5/genotype) (Suppl. Figure 10d), and looked into the distribution of the proteins identified with significant changes in 5XFAD groups. Most of the proteins that presented significant changes amongst 5XFAD groups did not show any differential distribution in the non-5XFAD paired analysis, despite being successfully detected (denoted as yellow dots in volcano plot, Suppl. Figure 10d).

Correspondence of the proteomic changes among populations was evaluated by correlating the fold change between comparisons using a triplot. The graph generated shows the protein abundance distribution measured using DIA-MS and its association with the mice genotypes (Fig. 4b). Assessing the proteins with different levels that were associated with enhanced phagocytosis in CD33m mice revealed increased levels of nestin in the comparisons between CD33m versus 5XFAD control and CD33M versus CD33m (Fig. 4b,c). Nestin is an intermediate filament associated with cell mobility/migration and has been reported to be upregulated in microglia under specific circumstances including activation states [50]. To exclude the possibility of neural stem cells (NSCs) contributing to nestin protein changes, we searched for markers of NSCs (SOX2, ABCG2, FABP7, NeuroD1, Hes1, Notch1) in our proteomics database. We only found ABCG2 and FABP7 at detectable levels, and neither was significantly different between the genotypes (Suppl. Figure 11).

Further immunostaining analysis of brain slices with an anti-nestin antibody clearly demonstrates that nestin protein levels were uniquely elevated in microglia-contact sites in CD33m mice at eight months (Fig. 4d). Total nestin signal was significantly higher in the CD33m + microglia (Fig. 4e). These results further support our earlier observations, regarding a higher density of PAM and enhanced microglia-plaque interface in CD33m.

Gene ontology (GO) enrichment analysis of the significantly changed proteins (Suppl. Figure 12) revealed alterations in pathways involved in inflammatory response, defective lipid metabolism, increased complement-dependent cytotoxicity, and cellular oxidative stress. The second comparison was done on CD33m versus 5XFAD control, wherein 40 proteins showed a significant increase in the CD33m, and 235 proteins were decreased compared to the 5XFAD control. Among the significant GO entries for the protein candidates, increased phagocytosis/engulfment, enhanced neuronal health and innervation, and improved lipid synthesis and storage were among the top biological processes with alterations. Finally, for the third comparison, CD33M versus CD33m, 17 increased proteins and 122 decreased proteins showed significant changes. Decreased phagocytosis or engulfment, along with disturbed cell adhesion, nerve morphogenesis and neuronal remodeling were consistently identified within the altered biological processes. We next did a more in-depth investigation by clustering list of identified proteins based on cell-type and exploring cell-specific changes in proteome (Suppl. Figure 13).

Comparative analysis of homeostatic (eg. P2Ry12 and Tmem119) and activatory (eg. CD9 and Lgals3) microglial markers suggested increased activation in CD33m^+^ microglia compared to control 5XFAD and CD33M^+^ cells (Suppl. Figure 13). A few other noteworthy identified hits in microglia cluster that showed higher abundance in CD33m^+^ microglia are Mtk1 (Metallothionein 1), associated with metabolic reprogramming in microglia [51], and Mdk (midkine), a neuroprotective growth factor involved in neuroimmune crosstalk [52, 53]. Additionally, CD33m^+^ microglia had decreased levels of proteins such as Bin2 (Bridging integrator 2) that is abundant in AD brains and associated with amyloid-driven neuroinflammation [54], and Rac2 (Rac family small GTPase-2) that is associated with detrimental effects of inflammatory response in microglia such as apoptosis [55]. Notably, focusing on other cell types we identified higher protein levels of Fgfr3 (Fibroblast growth factor receptor 3) in astrocytes of CD33M^+^ mice, which is associated with detrimental effects [56]. We also observed increased levels of two neuronal protein in CD33M^+^ mice, Arc (Activity-regulated cytoskeleton-associated protein) that has been shown to be abundant in the AD brain [57], and Chrm2 (Cholinergic Muscarinic Receptor 2), which highlights overactivation and damage of cholinergic neurons [58].

### Transcriptional profiling of microglia supports distinct effects of hCD33 isoforms

**Fig. 5:**
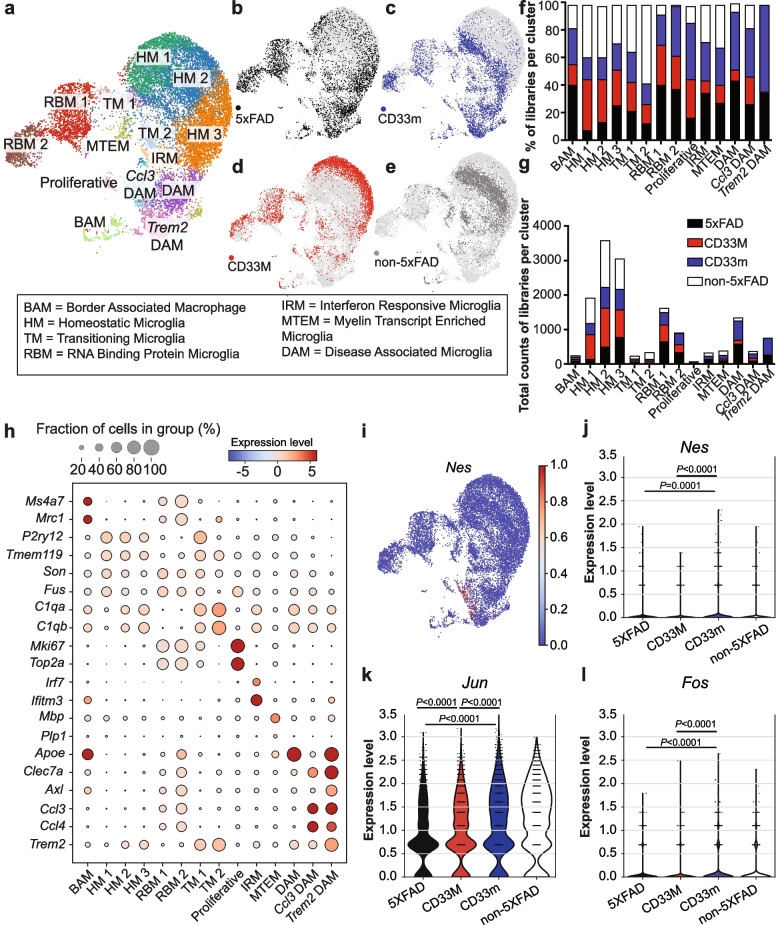
Single-cell RNA sequencing reveals *Ccl3* and *Trem2* DAM enriched in the CD33m genotype. **a** Unsupervised and iterative machine-learning based clustering of 15,200 microglia (*Hexb*^+^*Fcrls*^+^*Tmem119*^+^*Sall1*^+^) and BAM (*Ms4a7*^+^*Mrc1*^+^*Lyve1*^+^*Timd4*^+^) collected from 5XFAD control, CD33M 5XFAD, CD33m 5XFAD, and control non-5XFAD mice. Microglia from three homeostatic (HM1-3), two transitioning (TM1-2), two RNA binding protein (RBM1-2) subpopulations along with the disease-enriched interferon responsive (IRM), myelin transcript enriched (MTEM), and disease associated (DAM) clusters.  **b-e **UMAPs of individual samples showing (**b**) 5XFAD control, **c** CD33m 5XFAD, **d** CD33M 5XFAD, and **e** control non-5xFAD. **f**,**g** Separation of each cluster by (**f**) proportion of cells and (**g**) absolute number of cells belonging to each genotype. DAM, specifically *Trem2* DAM, are enriched in the CD33m group and reduced in the CD33M group. **h** Differential gene expression per cluster used to define microglial subpopulations. HM are characterized by homeostatic genes (*P2ry12, Tmem119*), RBM by genes related to RNA binding (*Son, Fus*), TM by a combination of homeostatic (*P2ry12, Tmem119*), complement (*C1qa, C1qb*), and proliferative (*MKi-67, Top2a*) genes. DAM are defined by *Clec7a* and *Apoe* expression and further delineated by expression of *Ccl3* and *Trem2*. **i** UMAP showing enriched *Nes* expression within the *Ccl3* DAM cluster. **j-l** Violin plots showing the upregulation of (**j**) *Nes*,  **k** *Jun*, and **l** *Fos* in the CD33m^+^ 5XFAD group relative to 5XFAD control and CD33M^+^ 5XFAD

To further explore the principles contributing to differences between hCD33 isoforms in modulating microglia response, we carried out single-cell RNA sequencing (scRNAseq) analysis on microglia extracted from the whole brain of 5XFAD mice from all three genotypes (2 male mice pooled per genotype) at eight months, along with age-matched non-5XFAD controls. After the quality control measures were carried out, the four datasets were pooled and clustered with Seurat V5, and single cell clustering assessment framework (SCCAF) was applied to re-cluster cells based on classic microglial gene expression of *Hexb, Fcrls, Sall1, Tmem119*, and *P2ry12*. Overall, we obtained 21,714 cells, which was reduced to 15,200 after quality control. Clustered cells were projected onto a uniform manifold approximation and projection (UMAP) to identify fourteen clusters, falling into six main categories: border associated macrophages (BAM), homeostatic microglia (HM), transitioning microglia (TM), RNA binding protein microglia (RBM), myelin transcript enriched microglia (MTEM), and disease associated microglia (DAM) (Fig. 5a). Three different integration methods applied to discard any potential batch effects preserved almost all of the major identified clusters (Suppl. Figure 14a-d). Moreover, evaluation of hCD33 transcript levels in all 5XFAD cohorts confirmed that at the transcript level, neither of the hCD33 transgene was overexpressed relative to mCd33 in CD33M^+^ 5XFAD and CD33m^+^ 5XFAD, which is in line with our previously reported values under homeostatic conditions (Suppl. Figure 15a,b) [15].

The four groups of mice (Fig. 5b-e) had a different distribution of cells in each of the subsets (Fig. 5f,g). Proliferative microglia expressed classic proliferative genes such as *Mki67* and *Top2a* (Fig. 5h). BAM are found in the meninges and perivascular space express markers such as *Mrc1* and *Ms4a7* [59–61]. We identified three, highly related, HM clusters enriched in 5XFAD CD33M. Adjacent to HM we found two clusters of microglia we referred to as TM, as they expressed higher levels of Trem2 than HM microglia. MTEM expressed non-microglial, oligodendrocyte lineage cell transcripts *Mbp* and *Plp1*. These are present despite quality control to remove doublets and have been identified by others using a 5XFAD model [62]. We find CD33M microglia are less prevalent in the MTEM cluster [63].

Based on expression of *Ifitm3* and *Irf7*, we identified an interferon responsive microglia (IRM) state. IRM have been shown to surround plaques and contribute to complement mediated synapse elimination [64, 65]. Here, we find that CD33m and 5XFAD controls are the predominant source of IRM, suggesting that IRM may support CD33m-promoting phagocytic activities.

We identified three different DAM subsets based on the expression of classic DAM markers *Clec7a* and *Axl,* which were absent in the non-5XFAD group (Fig. 5f,g). In addition to these classic DAM markers, we identified a DAM cluster specifically enriched for the chemokines *Ccl3 and Ccl4.* These factors were recently described to be released by microglia and act on neuronal Ccr5 to disrupt autophagy [66]. CD33m microglia were enriched within *Ccl3* DAM, and a DAM population enriched for these genes, but also expressing heightened levels of *Trem2* (termed *Trem2* DAM). The enrichment of CD33M^+^ microglia in HM clusters and reduction of CD33M microglia in DAM clusters is reminiscent of mice lacking TREM2 [67]. Given that DAM express genes related to phagocytosis such as *Cd86*, *ApoE*, *Lpl*, and *Cst7,* these data are consistent with heightened phagocytosis in CD33m^+^ microglia. To follow up these findings, we performed pseudobulk comparisons between the genotypes and consistently observed higher levels of DAM in CD33m^+^ microglia compared to CD33M (Suppl. Figure 16a). To decipher the detailed overall changes in each genotype, we performed GO enrichment on top DEGs (Suppl. Figure 16b). These analyses pointed to enhanced metabolic fitness, phagocytosis, and cell migration in CD33m^+^ microglia. CD33M^+^ microglia presented defects in neuroimmune responsiveness, cell mobility and migration, and cell activation. We also examined the specific expression levels of several genes including *Apoe*, *Clec7a*, *Tmeme119, Rac2, and Mki67* (Suppl. Figure 16c). Expression of the gene encoding nestin (*Nes*) was specific to DAM (Fig. 5i) and, in line with the proteomics data, its transcript levels were statistically higher in CD33m^+^ microglia (Fig. 5j). As activated, nestin^+^ microglia have been reported to have high levels of other intermediate filament components such as vimentin (*Vim*) as well as NG2 (*Cspg4*) [68, 69], we examined the expression level of these genes in our datasets as and observed highest levels of expression in CD33m^+^ (Suppl. Figure 16d).

We next carried out orthogonal validation on some of our scRNAseq, and specifically looked at Clec7a protein—a DAM marker [70, 71]—by IF staining of PAM (Suppl. Figure 17a). We found similar changes and saw significantly higher levels of Clec7a in CD33m^+^ mice compared to the other groups (Suppl. Figure 17a,b).

We also identified two clusters of microglia enriched for RNA binding proteins (termed RBM1 and RBM2), such as *Fus* and *Son*. RBM1 and RBM2 expressed low levels of DAM markers, suggesting they are DAM-like, while RBM2 expressed higher levels of *Apoe*, indicating this was a more reactive microglial population. We found no overt differences between CD33M and CD33m in RBM. Moreover, consistent with our previous report in homeostatic conditions [15], we observed upregulation of the immediate early genes (IEGs), such as *Jun*, and *Fos*, in 5XFAD-CD33m microglia (Fig. 5k,l). Fos is recruited to enhancers of scavenging and pathogen recognition receptors in microglia [72], which is consistent with *Fos* upregulation representing a more vigilant state.

### hCD33 isoforms distinctively affect neuronal health and behavior

**Fig. 6:**
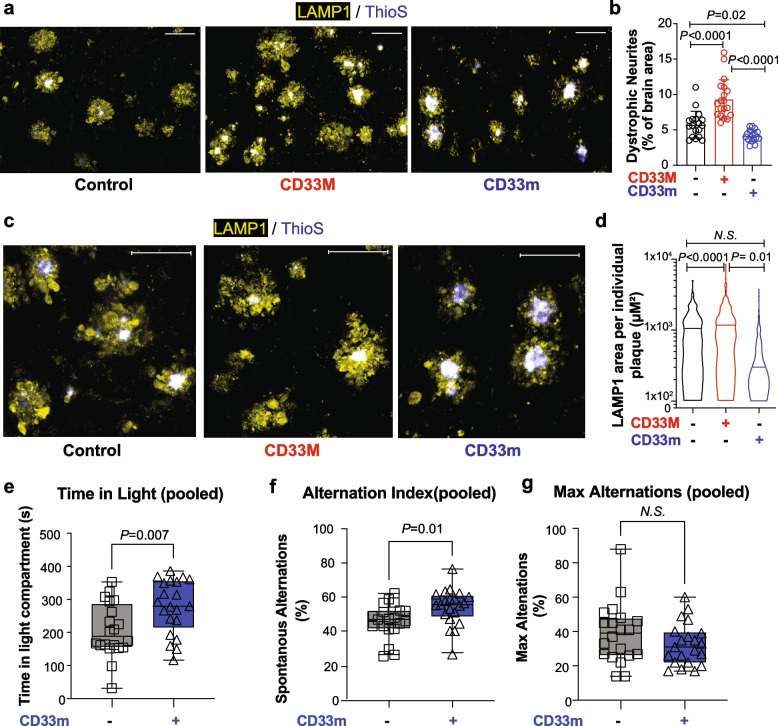
hCD33 isoforms impact neuronal health and animal behavior. **a** Representative immunofluorescent confocal images of dystrophic neurites stained with lysosomal marker LAMP1 (yellow) in the dorsal subiculum of control, CD33M, and CD33m mice at 8 months. Scale bar = 50 µm.** b** Quantification of total area of DNs in the dorsal subiculum. A total of 5 male and 5 female mice per genotype were used. **c** Higher magnification immunofluorescent confocal images of individual DNs in the dorsal subiculum of control, CD33M and CD33m mice at 8 months. Scale bar = 50 µm. **d** Quantification of DN area in individual neuritic plaques of 5XFAD control, CD33M, and CD33m mice at 8 months. Individual neuritic plaques within the subiculum and parts of frontal cortex adjacent to the subiculum were analyzed. A total of 300 plaques from 5 males and 5 females per genotype were quantified. **e** Quantification of time spent in light from a Light–Dark box assay carried out on 5XFAD and CD33m^+^ mice at 8 months (*n* = 13 female and 7 male mice for the 5XFAD control group, and *n* = 11 female and 10 male mice for the CD33m^+^ 5XFAD group were used). **f** Quantification of alternation index from a spontaneous Y-maze assay carried out on 5XFAD control and CD33m^+^ 5XFAD mice at 8 months (*n* = 13 female and 7 male mice for the 5XFAD control group, and *n* = 11 female and 10 male mice for the CD33m^+^ 5XFAD group were used). **g** Quantification of maximum alternation from the spontaneous Y-maze carried out on 5XFAD control and CD33m^+^ 5XFAD mice at 8 months

The substantial impact of hCD33 isoforms on total Aβ levels, as well as plaque composition motivated us to further explore their effects on neuronal health and neurobehavior of the mice. GO enrichment analysis gave a hint of enhanced neuronal health in CD33m mice compared to 5XFAD control (Suppl. Figure 12 and 13). Additionally, we analyzed dystrophic neurites (DNs) by staining for LAMP1, a marker of lysosomal vesicles in swollen neuritic plaques [73, 74]. Quantification of DNs within the subiculum and parts of the frontal cortex adjacent to the subiculum, further supported the detrimental and protective effects of CD33M and CD33m, respectively. CD33M mice had significantly higher levels of dystrophic neurites while CD33m mice had lower levels compared to the 5XFAD control group (Fig. 6a,b). Quantification of individual DNs revealed that CD33M mice had on average larger DNs compared to the two other groups (Fig. 6c,d). Moreover, the smaller neuritic plaques in CD33m had a higher number of PAM compared to both 5XFAD control and CD33M groups (Suppl. Figure 18a,b). Quantification of diffuse versus compact deposits in neuritic plaques revealed less than 40% of DNs constituting diffuse plaques in all genotypes (Suppl. Figure 18c) and there were no significant differences between groups in this regard. Correlation analysis on area of DN (LAMP1) and microglia (Iba1) pointed to significant correlation in 5XFAD control mice and no specific correlations in the CD33M or CD33m groups (Suppl. Figure 18d-f). Finally, correlation analysis on LAMP1^+^ area versus Aβ area within each DN pointed to significant positive correlation in all genotypes assessed separately (Suppl. Figure 18 g-i) and combined (Suppl. Figure 18j).

We initially performed a trial open-field test on a small cohort of 5XFAD female mice (5XFAD control, CD33M, and CD33m) at 1-year of age. This behavioral test has been previously used to identify altered cognitive performance associated with anxiety and exploratory behavior in 5XFAD mice [75]. CD33m^+^ mice spent significantly more time in the center compared to other groups without any differences in the other parameters, such as total distance and average velocity in the whole arena and the center (Suppl. Figure 19a-d), suggesting decreased anxiety and improved neurobehavioral outcomes in these animals compared to CD33M and 5XFAD control group. To better compare these results to the rest of our pathological analysis at 8 months, we repeated these studies on a larger cohort of male and female mice, focusing on 5XFAD control and CD33m 5XFAD groups to assess the cognitive outcomes from the gain-of-function effect of CD33m. At eight months, there were no significant differences between the two groups in the open-field test (Suppl. Figure 19e-h). Therefore, we carried out a light − dark box experiment as a more robust measurement of anxiety in mice [76, 77], and found that microglial expression of CD33m reduced anxiety in the 5XFAD background (Fig. 6e). This effect was specific to males, yet it remained significant in pooled mice of both sexes (Suppl. Figure 19i,j). We also tested the spatial working memory in these mice at the same time point, using the spontaneous Y-maze assay [78, 79], and found increased alternation index in the CD33m^+^ mice compared to the 5XFAD control (Fig. 6f). This effect was specific to males, yet it remained significant in pooled mice of both sexes (Suppl. Figure 19 k,l). There was no significant difference in total alternations between groups (Fig. 6g), suggesting that the significant increase in alternation index observed in the CD33m group is not due to changes in locomotor activity.

## Discussion

Numerous studies have linked polymorphisms in *CD33* to AD [8, 9, 80, 81], yet the exact function of the expressed protein in microglia is poorly understood. Studying the role of hCD33 in vivo is confounded by the fact that mCD33 is functionally divergent compared to hCD33 and, more importantly, does not generate an equivalent CD33m protein isoform [18, 19, 82]. Accordingly, mouse models of AD miss the CD33 component of human-specific microglial cell biology. Herein, leveraging Tg mice expressing either of the hCD33 isoforms in the microglial cell lineage, we explored the impact of each isoform and discovered profound and opposite effects of hCD33 isoforms in the context of aggressive amyloid deposition. Specifically, CD33M, preferentially encoded by the rs12459419C AD-susceptible allele in humans, exacerbated Aβ deposition and resulted in decreased plaque compaction. Conversely, CD33m, preferentially encoded by the rs12459419T AD-susceptible allele, decreased Aβ deposition and increased plaque compaction. These results point to a combination of loss-of-function and gain-of-function effects delivered by CD33m. Accordingly, there are two beneficial effects that come from the rs12459419T *CD33* allele: decreased CD33M expression and increased CD33m expression [83]. Based on a CD33-null allele (rs201074739) lacking any AD-protective effects [14, 84], the loss-of-function alone may be insufficient to produce an AD-protective effect, suggesting the gain-of-function role is at play for beneficial effects of rs12459419T.

Differences in CD33m mice, manifested as decreased total Aβ levels, enhanced plaque compaction, and higher number of PAM were consistently significant at both four and eight months timepoints. In contrast, changes in Aβ deposition and microglial cell response in CD33M took longer to develop, reaching significance only at eight months. Accordingly, in our models the overall impact of CD33m on modulating microglia and Aβ deposition is stronger and more profound compared to CD33M. This is in agreement with our previous work, wherein we reported that the in vitro gain-of-function role of CD33m is dominant over the suppressive effect of CD33M [15]. Regardless, the timing of events in our datasets reinforce the idea that hCD33 isoforms play unique and divergent roles in microglia.

CD33M mice presented higher levels of deposited Aβ compared to other genotypes, and the plaques in these mice were less compact and more diffuse in nature. Moreover, IF and scRNAseq analyses pointed to decreased numbers of PAM and DAM in CD33M^+^ microglia, which could account for less compaction. There are several possibilities on how CD33M may hinder DAM formation. Decreased DAM could be a secondary, long-term, consequence of impaired phagocytosis in CD33M^+^ microglia, which leads to elevated Aβ deposition. Another possibility is that CD33M directly impairs transcriptional reprogramming of microglia and CD33M is proposed to negatively regulate TREM2 [85, 86], which could be a mechanistic link. In this regard, CD33M microglia appear to be stalled at homeostatic (HM) and transitioning (TM) stages and fail to transition into DAM, which is reminiscent of mice lacking functional TREM2 [87]. A third possibility is that CD33M^+^ microglia initially differentiate into DAM but are driven into an alternative pro-inflammatory, detrimental, state due to overt activation and high proliferation [88]. Consistent with this idea, CD33M mice had increased Iba1 levels and higher levels of Ki-67^+^ microglia when normalized to PAM density. Higher Iba1 levels are in line with a prior report of increased Iba1 density from human AD patients homozygous for the AD-risk (rs12459419C) *CD33* allele [13]. In both our CD33M mice and this analysis of AD patients, higher Iba1 density may be a secondary consequence of elevated Aβ levels that leads to overly activated, yet less functional microglia. Indeed, constitutively activated microglia are detrimental and defective in the context of AD pathogenesis [88, 89].

We previously showed that the ability of CD33M to repress phagocytosis is dependent on its cytosolic ITIM [19], therefore, its role as a cell surface inhibitory receptor is likely a key contributing factor to the phenotypic outcomes we observe. It is interesting to speculate the potential role *trans* ligands of CD33M could have in vivo*.* This is especially worth exploring in the context of AD brain, as dramatic changes have been observed in the brain glycome of animal models of AD as well as human patients [90–92]*.* Recent advances in understanding human sialome and elucidation of the glycan ligands of CD33 [93–96] could be instrumental in spatial characterization of glycan ligands of CD33M at both regional and cellular levels in the brain, their changes during the course of disease, and their potential role in modulating microglia.

The decrease in total Aβ levels and increased plaque compaction in CD33m compared to 5XFAD control mice further support a gain-of-function role for CD33m in vivo. CD33m^+^ microglia had higher levels of internalized Aβ, which is in line with the ability of CD33m to enhance phagocytosis as we have previously reported [15]. Additionally, the remaining Aβ deposits in CD33m^+^ mice are of higher compaction, as evidenced by the decreased susceptibility to PK digestion within the insoluble fraction, as well as plaque compaction analysis by IF imaging. In summary, the functional changes in CD33m^+^ microglia, such as enhanced phagocytosis of Aβ, has the potential to modulate plaque composition simultaneously by removing Aβ deposits and enhancing compaction of remaining aggregated species [97, 98]. Moreover, CD33m enhanced the number of PAM and increased microglial plaque encapsulation. Although scRNAseq analysis did not show dramatically enhanced differences in the number of DAM between CD33m and 5XFAD control mice, fewer Aβ plaques in CD33m mice necessarily means a greater number of DAM per Aβ cluster in CD33m. Indeed, higher density of Clec7a staining in PAM strongly suggests that CD33m had a higher number of DAM per Aβ cluster.

Phagocytic clearance of small and intermediate Aβ assemblies can modify plaque composition in numerous ways such as discarding diffused Aβ that extend from the plaque core [46, 47, 99]. Therefore, enhanced phagocytic activity of CD33m^+^ microglia may be a key contributing factor to the AD protective effects of this isoform. However, there are no clear connections between enhanced phagocytosis and higher number of PAM/DAM, suggesting that the beneficial effects of CD33m may indeed be multifactorial. GO enrichment pathway analysis on our proteomics datasets, as well as top DEG scRNAseq libraries pointed to enhanced phagocytosis. In-depth investigation of proteome changes contributing to these pathways identified significant changes in nestin protein levels. Of note, the proteomics analysis in this study was performed on whole brain tissue and we further validated changes in microglial nestin levels by other techniques. Specifically, the changes in nestin were observed in the scRNAseq analysis of extracted microglia, as well as confocal IF analysis, wherein nestin was localized to microglia-plaque contact sites. Nestin is an intermediate filament abundantly expressed in cells with high migration capacity such as stem cells and is not normally expressed within homeostatic microglia. However, nestin has been shown to be upregulated in microglia under three circumstances: (i) following depletion of microglia when microglia are repopulating the brain [100], (ii) during inflammatory response in glial cells including activated microglia/macrophages [50], and (iii) in neonatal stages [101]. Moreover, nestin^+^ microglia in the brain have high levels of vimentin and neuron-glial antigen 2 (Ng2), both of which showed significantly higher transcript levels in scRNAseq. These results, combined with previous reports on upregulation of nestin in microglia under specific circumstances including activation states [50], support enhanced mobility and/or migration of CD33m^+^ microglia in response to amyloid deposition in 5XFAD. We cannot exclude other possible factors such as enhanced neurogenesis and increased NSC population driven by microglial cytokines additionally contributing to nestin protein changes in the whole brain, [91, 102]. However, we did not observe any significant differences in the protein levels of several NSC markers between genotypes.

In common with all these conditions is cellular migration and, consistent with this concept, a previous study in cultured BV2 microglia expressing CD33m reported enhanced migration [16]. Beyond enhancing migration/retention of microglia to plaques, it cannot be ruled out that CD33m^+^ microglia also enhanced transcriptional reprogramming of microglia to a DAM phenotype. CD33m^+^ microglia from 5XFAD mice had upregulated IEGs in accordance with our previous observation under homeostatic conditions [15], although it is not obvious whether these relate to DAM formation. Concurrently, a functional role for CD33m could also be at play, as we have previously shown that the in vitro gain-of-function role for CD33m was dependent on its C-terminal cytosolic signaling motifs [15]. In this regard, it is interesting to speculate that the gain-of-function role of CD33m is governed by its unique, intracellular localization, reported by our group and others [15, 102].

Our data suggest that the impact of hCD33 isoforms goes beyond affecting total Aβ levels or plaque compaction, and affects the overall state of other brain cells such as astrocytes and neurons. Specifically, changes in neuronal health were supported by: (i) differences in the size and number of dystrophic neurites; (ii) a GO signature associated with protected neural synaptic health in the mass spectrometry profiling; and (iii) behavior in the open-field test. These effects are in line with many other studies pointing to the positive, neuroprotective impacts of an efficient microglial response upon neurodegeneration, especially at early stages of disease wherein microglia directly engage in clearance of neurotoxic Aβ deposits [103, 104]. Specifically, it has been shown that microglia limit plaque expansion by remodeling plaque morphology, through phagocytic trimming of diffused Aβ deposits that extend from the plaque core, making plaques more compact [46, 105]. Moreover, microglia envelop the plaque with their processes and form a physical barrier to block accumulation of neurotoxic, Aβ assemblies and stop their spread [45, 106]. Differences in plaque compaction have been shown to be associated with higher levels of Aβ induced neurotoxicity and worsened axonal pathology in humans as well as mouse models of amyloidosis [45, 46, 73, 107]. In this regard, investigating the impact of CD33 in a double pathology model that can recapitulate both the plaques and tangle pathologies, will be an important next step.

Interest in modulating microglial cell response as a therapeutic approach in AD has been gaining momentum [5, 108]. Specifically, approaches focusing on modification of identified susceptibility variants are of great interest. For example, agonistic anti-TREM2 antibodies have shown promising effects in pre-clinical mouse models [109–112]. Anti-CD33 antibodies targeting the extracellular domain of CD33M had also entered phase I clinical trials but stopped for unknown reasons. Although immunotherapy-based approaches can be considered useful for targeting AD risk variants, findings from our previous work and current study highlights the importance of considering both hCD33 protein isoforms in the context of therapeutic targeting of the *CD33* loci. In other words, an approach that only considers CD33M will miss the gain-of-function effect of CD33m, which are clearly stronger based on our observations. In this regard, it will be important to re-consider strategies that skew the formation of different hCD33 isoforms, such as splicing modulators [113]. A better understanding of the mechanistic aspect of CD33m gain-of-function is extremely crucial, as discovering the precise mechanism(s) will help open the door to new, promising therapeutic targets. An unexplored question is whether the gain-of-function role for CD33m converges on downstream effects of TREM2. In this regard, some of the transcriptional changes in CD33m mice, such as increased expression of several IEGs, resemble reported observations in mice treated with agonistic anti-TREM2 antibody [112]. Therefore, establishing whether enhancing CD33m expression converges on TREM2 and potentially drives synergistic effects with TREM2 agonism is a unique point to address.

The hCD33 Tg mouse models used in this study were generated with the aim to decipher the functional impact of individual isoforms in the context of Aβ pathogenesis. Although our findings clearly show a detrimental impact from CD33M and provides in vivo evidence towards protective, beneficial effects of CD33m it should be noted that the *CD33* gene, whether it is the rs12459419T or C allele, have both isoforms expressed. Using the same transgenic mice under homeostatic conditions, we previously showed that the gain-of-function role for CD33m in microglia is dominant over the suppressive effects of CD33M [15]. However, it is possible that the combination of the two isoforms could produce a phenotype in AD that is distinct from ones that can be generated for either isoform on its own. Additionally, while the focus of these studies was hCD33 isoforms, due to the significant differences between hCD33 and mCD33, we cannot fully rule out an effect from mCD33.

## Conclusions

Deciphering the functional aspect of AD susceptibility factors can lead to identification of pathways that can potentially be targeted for development of novel, promising therapeutics. The AD-protective effects of a minor *CD33* SNP motivated us to create a platform and independently investigate the loss-of-function and gain-of-function roles for the two CD33 protein isoforms associated with AD risk. Overall, our results suggest that AD susceptibility mediated by the *CD33* loci is a complex phenotype stemming from independent and opposite effects of hCD33 isoforms on modulating the microglial cell response to Aβ deposition. The divergent roles of hCD33 isoforms in microglia have an impact on total Aβ levels and plaque composition followed by downstream effects on neuronal health and cognitive function of the animals.

### Supplementary Information

Supplementary Material 1.

## Data Availability

The RNA-seq expression data has been deposited to the GEO database. The full list of mass spectrometry data including peptides, and substrates identified is provided online (.xlsx) and also deposited in the MASSIVE repository.

## Abbreviations

AD: Alzheimer’s disease
Siglec: Sialic acid-binding immunoglobulin-type lectin
hCD33: Human CD33 protein
mCD33: Murine/mouse CD33 protein
CD33M: Human CD33 major isoform
CD33m: Human CD33 minor isoform
GWAS: Genome-wide association studies
SNP: Single nucleotide polymorphism
Aβ: Amyloid beta
ITIM: Immunoreceptor tyrosine-based inhibitory motif
ITAM: Immunoreceptor tyrosine-based activatory motif
IF: Immunofluorescence microscopy
FOV: Field of View
SCCAF: Single-Cell Clustering Assignment Framework
pySCENIC: Python Single-Cell rEgulatory Network Inference and Clustering
UMAP: Uniform manifold approximation and projection
IEG: Immediate early gene
scRNAseq: Single cell RNA sequence
DEGs: Differentially expressed genes
DAM: Disease associated microglia
DIA-MS: Data-independent acquisition mass spectrometry
PAM: Plaque associated microglia
BAM: Border associated macrophages
TM: Transitioning microglia
RBM: RNA binding protein microglia
HM: Homeostatic microglia
GO: Gene ontology

## References

[1] Ballard C, Gauthier S, Corbett A, Brayne C, Aarsland D, Jones E (2011). **Alzheimer's disease.**
*the*. Lancet.

[2] Lane CA, Hardy J, Schott JM (2018). Alzheimer's disease European journal of neurology.

[3] Rezaie P, Male D (2002). Mesoglia & microglia–a historical review of the concept of mononuclear phagocytes within the central nervous system. J Hist Neurosci.

[4] Lee CD, Landreth GE (2010). The role of microglia in amyloid clearance from the AD brain. J Neural Transm.

[5] Sarlus H, Heneka MT (2017). Microglia in Alzheimer’s disease. J Clin Investig.

[6] Salter MW, Stevens B (2017). Microglia emerge as central players in brain disease. Nat Med.

[7] Jones L, Holmans PA, Hamshere ML, Harold D, Moskvina V, Ivanov D, Pocklington A, Abraham R, Hollingworth P, Sims R (2010). Genetic evidence implicates the immune system and cholesterol metabolism in the aetiology of Alzheimer's disease. PLoS ONE.

[8] Hollingworth P, Harold D, Sims R, Gerrish A, Lambert J-C, Carrasquillo MM, Abraham R, Hamshere ML, Pahwa JS, Moskvina V (2011). Common variants at ABCA7, MS4A6A/MS4A4E, EPHA1, CD33 and CD2AP are associated with Alzheimer's disease. Nat Genet.

[9] Naj AC, Jun G, Beecham GW, Wang L-S, Vardarajan BN, Buros J, Gallins PJ, Buxbaum JD, Jarvik GP, Crane PK (2011). Common variants at MS4A4/MS4A6E, CD2AP, CD33 and EPHA1 are associated with late-onset Alzheimer's disease. Nat Genet.

[10] Hayden KM, Lutz MW, Kuchibhatla M, Germain C, Plassman BL (2015). Effect of APOE and CD33 on cognitive decline. PLoS ONE.

[11] Malik M, Simpson JF, Parikh I, Wilfred BR, Fardo DW, Nelson PT, Estus S (2013). CD33 Alzheimer's risk-altering polymorphism, CD33 expression, and exon 2 splicing. J Neurosci.

[12] Raj T, Ryan KJ, Replogle JM, Chibnik LB, Rosenkrantz L, Tang A, Rothamel K, Stranger BE, Bennett DA, Evans DA (2014). CD33: increased inclusion of exon 2 implicates the Ig V-set domain in Alzheimer's disease susceptibility. Hum Mol Genet.

[13] Bradshaw EM, Chibnik LB, Keenan BT, Ottoboni L, Raj T, Tang A, Rosenkrantz LL, Imboywa S, Lee M, Von Korff A (2013). CD33 Alzheimer's disease locus: altered monocyte function and amyloid biology. Nat Neurosci.

[14] Estus S, Shaw BC, Devanney N, Katsumata Y, Press EE, Fardo DW (2019). Evaluation of CD33 as a genetic risk factor for Alzheimer’s disease. Acta Neuropathol.

[15] Bhattacherjee A, Jung J, Zia S, Ho M, Eskandari-Sedighi G, St. Laurent CD, McCord KA, Bains A, Sidhu G, Sarkar S (2021). The CD33 short isoform is a gain-of-function variant that enhances Aβ 1–42 phagocytosis in microglia. Mol Neurodegener.

[16] Butler CA, Thornton P, Brown GC (2021). CD33M inhibits microglial phagocytosis, migration and proliferation, but the Alzheimer’s disease-protective variant CD33m stimulates phagocytosis and proliferation, and inhibits adhesion. J Neurochem.

[17] Schwarz F, Springer SA, Altheide TK, Varki NM, Gagneux P, Varki A (2016). Human-specific derived alleles of CD33 and other genes protect against postreproductive cognitive decline. Proc Natl Acad Sci.

[18] Eskandari-Sedighi G, Jung J, Macauley MS. CD33 isoforms in microglia and Alzheimer's disease: Friend and foe. Mol Asp Med*.* 2022:101111. 10.1016/j.mam.2022.101111 35940942

[19] Bhattacherjee A, Rodrigues E, Jung J, Luzentales-Simpson M, Enterina JR, Galleguillos D, St. Laurent CD, Nakhaei-Nejad M, Fuchsberger FF, Streith L. Repression of phagocytosis by human CD33 is not conserved with mouse CD33. *Commun Biol.* 2019, 2:450. 10.1038/s42003-019-0698-6 PMC6890642 31815204

[20] Oakley H, Cole SL, Logan S, Maus E, Shao P, Craft J, Guillozet-Bongaarts A, Ohno M, Disterhoft J, Van Eldik L (2006). Intraneuronal β-amyloid aggregates, neurodegeneration, and neuron loss in transgenic mice with five familial Alzheimer's disease mutations: potential factors in amyloid plaque formation. J Neurosci.

[21] Duan S, Koziol-White CJ, Jester WF, Smit SA, Nycholat CM, Macauley MS, Panettieri RA, Paulson JC (2021). CD33 recruitment inhibits IgE-mediated anaphylaxis and desensitizes mast cells to allergen. J Clin Investig.

[22] Schmidt ML, Robinson KA, Lee V, Trojanowski JQ (1995). Chemical and immunological heterogeneity of fibrillar amyloid in plaques of Alzheimer's disease and Down's syndrome brains revealed by confocal microscopy. Am J Pathol.

[23] Santarriaga S, Luecke I, Ebert AD. Detection of soluble and insoluble protein species in patient-derived iPSCs. In *Stem Cell Assays: Methods and Protocols.* Springer; 2022: 73–84 10.1007/978-1-0716-1979-7_6 35507156

[24] Crowell AM, MacLellan DL, Doucette AA (2015). A two-stage spin cartridge for integrated protein precipitation, digestion and SDS removal in a comparative bottom-up proteomics workflow. J Proteomics.

[25] Mehta D, Scandola S, Uhrig RG (2022). BoxCar and Library-Free Data-Independent Acquisition Substantially Improve the Depth, Range, and Completeness of Label-Free Quantitative Proteomics. Anal Chem.

[26] Sobue A, Komine O, Hara Y, Endo F, Mizoguchi H, Watanabe S, Murayama S, Saito T, Saido TC, Sahara N (2021). Microglial gene signature reveals loss of homeostatic microglia associated with neurodegeneration of Alzheimer’s disease. Acta Neuropathol Commun.

[27] Hammond TR, Dufort C, Dissing-Olesen L, Giera S, Young A, Wysoker A, Walker AJ, Gergits F, Segel M, Nemesh J (2019). Single-cell RNA sequencing of microglia throughout the mouse lifespan and in the injured brain reveals complex cell-state changes. Immunity.

[28] Wolf FA, Angerer P, Theis FJ (2018). SCANPY: large-scale single-cell gene expression data analysis. Genome Biol.

[29] Raudvere U, Kolberg L, Kuzmin I, Arak T, Adler P, Peterson H, Vilo J (2019). g:Profiler: a web server for functional enrichment analysis and conversions of gene lists (2019 update). Nucleic Acids Res.

[30] Bhattacharya S, Haertel C, Maelicke A, Montag D (2014). Galantamine slows down plaque formation and behavioral decline in the 5XFAD mouse model of Alzheimer’s disease. PLoS ONE.

[31] Guo L, Zhong MB, Zhang L, Zhang B, Cai D (2022). Sex differences in Alzheimer’s disease: Insights from the multiomics landscape. Biol Psychiat.

[32] Oblak AL, Lin PB, Kotredes KP, Pandey RS, Garceau D, Williams HM, Uyar A, O’Rourke R, O’Rourke S, Ingraham C (2021). Comprehensive evaluation of the 5XFAD mouse model for preclinical testing applications: a MODEL-AD study. Frontiers in aging neuroscience.

[33] Spangenberg E, Severson PL, Hohsfield LA, Crapser J, Zhang J, Burton EA, Zhang Y, Spevak W, Lin J, Phan NY (2019). Sustained microglial depletion with CSF1R inhibitor impairs parenchymal plaque development in an Alzheimer’s disease model. Nat Commun.

[34] Delizannis AT, Nonneman A, Tsering W, De Bondt A, Van den Wyngaert I, Zhang B, Meymand E, Olufemi MF, Koivula P, Maimaiti S (2021). Effects of microglial depletion and TREM2 deficiency on Aβ plaque burden and neuritic plaque tau pathology in 5XFAD mice. Acta Neuropathol Commun.

[35] Bussière T, Bard F, Barbour R, Grajeda H, Guido T, Khan K, Schenk D, Games D, Seubert P, Buttini M (2004). Morphological characterization of Thioflavin-S-positive amyloid plaques in transgenic Alzheimer mice and effect of passive Aβ immunotherapy on their clearance. Am J Pathol.

[36] D’Andrea M, Nagele R (2010). Morphologically distinct types of amyloid plaques point the way to a better understanding of Alzheimer’s disease pathogenesis. Biotech Histochem.

[37] DeTure MA, Dickson DW (2019). The neuropathological diagnosis of Alzheimer’s disease. Mol Neurodegener.

[38] Dumurgier J, Mouton-Liger F, Lapalus P, Prevot M, Laplanche J-L, Hugon J, Paquet C (2013). Network GdIdLCS: **Cerebrospinal fluid PKR level predicts cognitive decline in Alzheimer’s disease**. PLoS ONE.

[39] Di Fede G, Catania M, Maderna E, Ghidoni R, Benussi L, Tonoli E, Giaccone G, Moda F, Paterlini A, Campagnani I (2018). Molecular subtypes of Alzheimer’s disease. Sci Rep.

[40] Langer F, Eisele YS, Fritschi SK, Staufenbiel M, Walker LC, Jucker M (2011). Soluble Aβ seeds are potent inducers of cerebral β-amyloid deposition. J Neurosci.

[41] Ruiz-Riquelme A, Mao A, Barghash MM, Lau HH, Stuart E, Kovacs GG, Nilsson KPR, Fraser PE, Schmitt-Ulms G, Watts JC (2021). Aβ43 aggregates exhibit enhanced prion-like seeding activity in mice. Acta Neuropathol Commun.

[42] Ulrich JD, Ulland TK, Mahan TE, Nystrom S, Nilsson KP, Song WM, Zhou Y, Reinartz M, Choi S, Jiang H (2018). ApoE facilitates the microglial response to amyloid plaque pathology. J Exp Med.

[43] Griciuc A, Serrano-Pozo A, Parrado AR, Lesinski AN, Asselin CN, Mullin K, Hooli B, Choi SH, Hyman BT, Tanzi RE (2013). Alzheimer’s disease risk gene CD33 inhibits microglial uptake of amyloid beta. Neuron.

[44] Perego C, Fumagalli S, De Simoni MG (2011). Temporal pattern of expression and colocalization of microglia/macrophage phenotype markers following brain ischemic injury in mice. J Neuroinflammation.

[45] Condello C, Yuan P, Schain A, Grutzendler J (2015). Microglia constitute a barrier that prevents neurotoxic protofibrillar Aβ42 hotspots around plaques. Nat Commun.

[46] Huang Y, Happonen KE, Burrola PG, O’Connor C, Hah N, Huang L, Nimmerjahn A, Lemke G (2021). Microglia use TAM receptors to detect and engulf amyloid β plaques. Nat Immunol.

[47] Venegas C, Kumar S, Franklin BS, Dierkes T, Brinkschulte R, Tejera D, Vieira-Saecker A, Schwartz S, Santarelli F, Kummer MP (2017). Microglia-derived ASC specks cross-seed amyloid-β in Alzheimer’s disease. Nature.

[48] Villacampa N, Sarlus H, Martorell P, Slutzkin I, Mcmanus RM, Beyer M, Segal E, Heneka MT (2021). Proliferating microglia exhibit unique transcriptomal and functional alterations in Alzheimer’s disease. Alzheimers Dement.

[49] Estfanous S, Daily KP, Eltobgy M, Deems NP, Anne MN, Krause K, Badr A, Hamilton K, Carafice C, Hegazi A (2021). Elevated expression of MiR-17 in microglia of Alzheimer’s disease patients abrogates autophagy-mediated amyloid-β degradation. Front Immunol.

[50] Krishnasamy S, Weng Y-C, Thammisetty SS, Phaneuf D, Lalancette-Hebert M, Kriz J (2017). Molecular imaging of nestin in neuroinflammatory conditions reveals marked signal induction in activated microglia. J Neuroinflammation.

[51] Gu C, Wang F, Zhang YT, Wei SZ, Liu JY, Sun HY, Wang GH, Liu CF (2021). Microglial MT1 activation inhibits LPS-induced neuroinflammation via regulation of metabolic reprogramming. Aging Cell.

[52] Guo X, Pan Y, Xiong M, Sanapala S, Anastasaki C, Cobb O, Dahiya S, Gutmann DH (2020). Midkine activation of CD8(+) T cells establishes a neuron-immune-cancer axis responsible for low-grade glioma growth. Nat Commun.

[53] Campbell WA, Fritsch-Kelleher A, Palazzo I, Hoang T, Blackshaw S, Fischer AJ (2021). Midkine is neuroprotective and influences glial reactivity and the formation of Muller glia-derived progenitor cells in chick and mouse retinas. Glia.

[54] Naj AC, Schellenberg GD (2017). Alzheimer's Disease Genetics C: **Genomic variants, genes, and pathways of Alzheimer's disease: An overview**. Am J Med Genet B Neuropsychiatr Genet.

[55] An M, Qiu Y, Wang C, Ma P, Ding Y (2023). Rac2 enhances activation of microglia and astrocytes, inflammatory response, and apoptosis via activating JNK signaling pathway and suppressing SIRT1 expression in chronic constriction injury-induced neuropathic pain. J Neuropathol Exp Neurol.

[56] Cornille M, Moriceau S, Khonsari RH, Heuze Y, Loisay L, Boitez V, Morice A, Arnaud E, Collet C, Bensidhoum M (2022). FGFR3 overactivation in the brain is responsible for memory impairments in Crouzon syndrome mouse model. J Exp Med..

[57] Kerrigan TL, Randall AD: A new player in the "synaptopathy" of Alzheimer's disease - arc/arg 3.1. *Front Neurol* 2013, 4:9. 10.3389/fneur.2013.00009 PMC3570765 23407382

[58] Ferreira-Vieira TH, Guimaraes IM, Silva FR, Ribeiro FM (2016). Alzheimer's disease: Targeting the Cholinergic System. Curr Neuropharmacol.

[59] Van Hove H, Martens L, Scheyltjens I, De Vlaminck K, Pombo Antunes AR, De Prijck S, Vandamme N, De Schepper S, Van Isterdael G, Scott CL (2019). A single-cell atlas of mouse brain macrophages reveals unique transcriptional identities shaped by ontogeny and tissue environment. Nat Neurosci.

[60] Mrdjen D, Pavlovic A, Hartmann FJ, Schreiner B, Utz SG, Leung BP, Lelios I, Heppner FL, Kipnis J, Merkler D (2018). High-Dimensional Single-Cell Mapping of Central Nervous System Immune Cells Reveals Distinct Myeloid Subsets in Health, Aging, and Disease. Immunity.

[61] Jordao MJC, Sankowski R, Brendecke SM, Sagar, Locatelli G, Tai YH, Tay TL, Schramm E, Armbruster S, Hagemeyer N (2019). Single-cell profiling identifies myeloid cell subsets with distinct fates during neuroinflammation. Science.

[62] Depp C, Sun T, Sasmita AO, Spieth L, Berghoff SA, Nazarenko T, Overhoff K, Steixner-Kumar AA, Subramanian S, Arinrad S (2023). Myelin dysfunction drives amyloid-beta deposition in models of Alzheimer's disease. Nature.

[63] Chen J-F, Liu K, Hu B, Li R-R, Xin W, Chen H, Wang F, Chen L, Li R-X, Ren S-Y (2021). Enhancing myelin renewal reverses cognitive dysfunction in a murine model of Alzheimer’s disease. Neuron.

[64] Roy ER, Wang B (2020). Wan Y-w, Chiu G, Cole A, Yin Z, Propson NE, Xu Y, Jankowsky JL, Liu Z: **Type I interferon response drives neuroinflammation and synapse loss in Alzheimer disease**. J Clin Investig.

[65] Roy ER, Chiu G, Li S, Propson NE, Kanchi R, Wang B, Coarfa C, Zheng H, Cao W (2022). Concerted type I interferon signaling in microglia and neural cells promotes memory impairment associated with amyloid β plaques. Immunity.

[66] Festa BP, Siddiqi FH, Jimenez-Sanchez M, Won H, Rob M, Djajadikerta A, Stamatakou E, Rubinsztein DC (2023). Microglial-to-neuronal CCR5 signaling regulates autophagy in neurodegeneration. Neuron.

[67] Deczkowska A, Keren-Shaul H, Weiner A, Colonna M, Schwartz M, Amit I (2018). Disease-associated microglia: a universal immune sensor of neurodegeneration. Cell.

[68] Wohl SG, Schmeer CW, Friese T, Witte OW, Isenmann S (2011). In situ dividing and phagocytosing retinal microglia express nestin, vimentin, and NG2 in vivo. PLoS ONE.

[69] Takamori Y, Mori T, Wakabayashi T, Nagasaka Y, Matsuzaki T, Yamada H (2009). Nestin-positive microglia in adult rat cerebral cortex. Brain Res.

[70] Krasemann S, Madore C, Cialic R, Baufeld C, Calcagno N, El Fatimy R, Beckers L (2017). O’loughlin E, Xu Y, Fanek Z: **The TREM2-APOE pathway drives the transcriptional phenotype of dysfunctional microglia in neurodegenerative diseases**. Immunity.

[71] Zia S, Hammond BP, Zirngibl M, Sizov A, Baaklini CS, Panda SP, Ho MF, Lee KV, Mainali A, Burr MK (2022). Single-cell microglial transcriptomics during demyelination defines a microglial state required for lytic carcass clearance. Mol Neurodegener.

[72] Han RT, Vainchtein ID, Schlachetzki JCM, Cho FS, Dorman LC, Ahn E, Kim DK, Barron JJ, Nakao-Inoue H, Molofsky AB (2023). Microglial pattern recognition via IL-33 promotes synaptic refinement in developing corticothalamic circuits in mice. J Exp Med..

[73] Meilandt WJ, Ngu H, Gogineni A, Lalehzadeh G, Lee S-H, Srinivasan K, Imperio J, Wu T, Weber M, Kruse AJ (2020). Trem2 deletion reduces late-stage amyloid plaque accumulation, elevates the Aβ42: Aβ40 ratio, and exacerbates axonal dystrophy and dendritic spine loss in the PS2APP Alzheimer's mouse model. J Neurosci.

[74] Sadleir KR, Kandalepas PC, Buggia-Prévot V, Nicholson DA, Thinakaran G, Vassar R (2016). Presynaptic dystrophic neurites surrounding amyloid plaques are sites of microtubule disruption, BACE1 elevation, and increased Aβ generation in Alzheimer’s disease. Acta Neuropathol.

[75] Seibenhener ML, Wooten MC: Use of the open field maze to measure locomotor and anxiety-like behavior in mice. *Journal of visualized experiments: JoVE* 2015. 10.3791/52434 PMC4354627 25742564

[76] Bourin M, Hascoet M (2003). The mouse light/dark box test. Eur J Pharmacol.

[77] Campos AC, Fogaca MV, Aguiar DC, Guimaraes FS (2013). Animal models of anxiety disorders and stress. Braz J Psychiatry.

[78] Kraeuter AK, Guest PC, Sarnyai Z (2019). The Y-Maze for Assessment of Spatial Working and Reference Memory in Mice. Methods Mol Biol.

[79] Lalonde R (2002). The neurobiological basis of spontaneous alternation. Neurosci Biobehav Rev.

[80] Bertram L, Lange C, Mullin K, Parkinson M, Hsiao M, Hogan MF, Schjeide BM, Hooli B, Divito J, Ionita I (2008). Genome-wide association analysis reveals putative Alzheimer's disease susceptibility loci in addition to APOE. The American Journal of Human Genetics.

[81] Karch CM, Jeng AT, Nowotny P, Cady J, Cruchaga C, Goate AM (2012). Expression of novel Alzheimer’s disease risk genes in control and Alzheimer’s disease brains. PLoS ONE.

[82] Brinkman-Van der Linden EC (2003). Angata T, Reynolds SA, Powell LD, Hedrick SM, Varki A: **CD33/Siglec-3 binding specificity, expression pattern, and consequences of gene deletion in mice**. Mol Cell Biol.

[83] Malik M, Chiles J, Xi HS, Medway C, Simpson J, Potluri S, Howard D, Liang Y, Paumi CM, Mukherjee S (2015). Genetics of CD33 in Alzheimer's disease and acute myeloid leukemia. Hum Mol Genet.

[84] Papageorgiou I, Loken MR, Brodersen LE, Gbadamosi M, Uy GL, Meshinchi S, Lamba JK (2019). CCGG deletion (rs201074739) in CD33 results in premature termination codon and complete loss of CD33 expression: another key variant with potential impact on response to CD33-directed agents. Leuk Lymphoma.

[85] Griciuc A, Patel S, Federico AN, Choi SH, Innes BJ, Oram MK, Cereghetti G, McGinty D, Anselmo A, Sadreyev RI (2019). TREM2 acts downstream of CD33 in modulating microglial pathology in Alzheimer’s disease. Neuron.

[86] Chan G, White CC, Winn PA, Cimpean M, Replogle JM, Glick LR, Cuerdon NE, Ryan KJ, Johnson KA, Schneider JA (2015). CD33 modulates TREM2: convergence of Alzheimer loci. Nat Neurosci.

[87] McQuade A, Kang YJ, Hasselmann J, Jairaman A, Sotelo A, Coburn M, Shabestari SK, Chadarevian JP, Fote G, Tu CH (2020). Gene expression and functional deficits underlie TREM2-knockout microglia responses in human models of Alzheimer’s disease. Nat Commun.

[88] Hu Y, Fryatt GL, Ghorbani M, Obst J, Menassa DA, Martin-Estebane M, Muntslag TA, Olmos-Alonso A, Guerrero-Carrasco M, Thomas D (2021). Replicative senescence dictates the emergence of disease-associated microglia and contributes to Aβ pathology. Cell Rep.

[89] Streit WJ, Braak H, Xue Q-S, Bechmann I (2009). Dystrophic (senescent) rather than activated microglial cells are associated with tau pathology and likely precede neurodegeneration in Alzheimer’s disease. Acta Neuropathol.

[90] Hawkinson TR, Clarke HA, Young LE, Conroy LR, Markussen KH, Kerch KM, Johnson LA, Nelson PT, Wang C, Allison DB (2022). In situ spatial glycomic imaging of mouse and human Alzheimer's disease brains. Alzheimers Dement.

[91] Peng W, Kobeissy F, Mondello S, Barsa C, Mechref Y: MS-based glycomics: An analytical tool to assess nervous system diseases. *Frontiers in Neuroscience* 2022, 16. 10.3389/fnins.2022.1000179 PMC9671362 36408389

[92] Suttapitugsakul S, Stavenhagen K, Donskaya S, Bennett DA, Mealer RG, Seyfried NT, Cummings RD (2022). Glycoproteomics Landscape of Asymptomatic and Symptomatic Human Alzheimer’s Disease Brain. Mol Cell Proteomics.

[93] Büll C, Nason R, Sun L, Van Coillie J, Madriz Sørensen D, Moons SJ, Yang Z, Arbitman S, Fernandes SM, Furukawa S (2021). Probing the binding specificities of human Siglecs by cell-based glycan arrays. Proc Natl Acad Sci.

[94] Gonzalez-Gil A, Porell RN, Fernandes SM, Maenpaa E, Li TA, Li T, Wong PC, Aoki K, Tiemeyer M, Zaikuan JY (2022). Human brain sialoglycan ligand for CD33, a microglial inhibitory Siglec implicated in Alzheimer’s disease. J Biol Chem.

[95] Jung J, Enterina JR, Bui DT, Mozaneh F, Lin P-H (2021). Nitin, Kuo C-W, Rodrigues E, Bhattacherjee A, Raeisimakiani P: **Carbohydrate sulfation as a mechanism for fine-tuning Siglec ligands**. ACS Chem Biol.

[96] Rodrigues E, Jung J, Park H, Loo C, Soukhtehzari S, Kitova EN, Mozaneh F, Daskhan G, Schmidt EN, Aghanya V (2020). A versatile soluble siglec scaffold for sensitive and quantitative detection of glycan ligands. Nat Commun.

[97] Huang Y, Happonen KE, Burrola PG, O'Connor C, Hah N, Huang L, Nimmerjahn A, Lemke G (2021). Microglia use TAM receptors to detect and engulf amyloid beta plaques. Nat Immunol.

[98] Venegas C, Kumar S, Franklin BS, Dierkes T, Brinkschulte R, Tejera D, Vieira-Saecker A, Schwartz S, Santarelli F, Kummer MP (2017). Microglia-derived ASC specks cross-seed amyloid-beta in Alzheimer's disease. Nature.

[99] Bassil R, Shields K, Granger K, Zein I, Ng S, Chih B (2021). Improved modeling of human AD with an automated culturing platform for iPSC neurons, astrocytes and microglia. Nat Commun.

[100] Huang Y, Xu Z, Xiong S, Sun F, Qin G, Hu G, Wang J, Zhao L, Liang Y-X, Wu T (2018). Repopulated microglia are solely derived from the proliferation of residual microglia after acute depletion. Nat Neurosci.

[101] Wlodarczyk A, Holtman IR, Krueger M, Yogev N, Bruttger J, Khorooshi R, Benmamar-Badel A, de Boer-Bergsma JJ, Martin NA, Karram K (2017). A novel microglial subset plays a key role in myelinogenesis in developing brain. EMBO J.

[102] Siddiqui SS, Springer SA, Verhagen A, Sundaramurthy V, Alisson-Silva F, Jiang W, Ghosh P, Varki A (2017). The Alzheimer's disease–protective CD33 splice variant mediates adaptive loss of function via diversion to an intracellular pool. J Biol Chem.

[103] Casali BT, MacPherson KP, Reed-Geaghan EG, Landreth GE (2020). Microglia depletion rapidly and reversibly alters amyloid pathology by modification of plaque compaction and morphologies. Neurobiol Dis.

[104] Spangenberg EE, Green KN (2017). Inflammation in Alzheimer’s disease: lessons learned from microglia-depletion models. Brain Behav Immun.

[105] Shabestari SK, Morabito S, Danhash EP, McQuade A, Sanchez JR, Miyoshi E, Chadarevian JP, Claes C, Coburn MA, Hasselmann J (2022). Absence of microglia promotes diverse pathologies and early lethality in Alzheimer’s disease mice. Cell Rep.

[106] Yuan P, Condello C, Keene CD, Wang Y, Bird TD, Paul SM, Luo W, Colonna M, Baddeley D, Grutzendler J (2016). TREM2 haplodeficiency in mice and humans impairs the microglia barrier function leading to decreased amyloid compaction and severe axonal dystrophy. Neuron.

[107] Walker LC: Aβ plaques. *Free neuropathology* 2020, 1. 10.17879/freeneuropathology-2020-3025 PMC7745791 33345256

[108] McQuade A, Blurton-Jones M (2019). Microglia in Alzheimer's disease: exploring how genetics and phenotype influence risk. J Mol Biol.

[109] Fassler M, Rappaport MS, Cuño CB, George J (2021). Engagement of TREM2 by a novel monoclonal antibody induces activation of microglia and improves cognitive function in Alzheimer’s disease models. J Neuroinflammation.

[110] Schlepckow K, Monroe KM, Kleinberger G, Cantuti-Castelvetri L, Parhizkar S, Xia D, Willem M, Werner G, Pettkus N, Brunner B (2020). Enhancing protective microglial activities with a dual function TREM 2 antibody to the stalk region. EMBO Mol Med.

[111] van Lengerich B, Zhan L, Xia D, Chan D, Joy D, Park JI, Tatarakis D, Calvert M, Hummel S, Lianoglou S (2023). A TREM2-activating antibody with a blood–brain barrier transport vehicle enhances microglial metabolism in Alzheimer’s disease models. Nat Neurosci.

[112] Wang S, Mustafa M, Yuede CM, Salazar SV, Kong P, Long H, Ward M, Siddiqui O, Paul R, Gilfillan S (2020). Anti-human TREM2 induces microglia proliferation and reduces pathology in an Alzheimer’s disease model. J Exp Med.

[113] van Bergeijk P, Seneviratne U, Aparicio-Prat E, Stanton R, Hasson SA (2019). SRSF1 and PTBP1 are trans-acting factors that suppress the formation of a CD33 splicing isoform linked to Alzheimer’s disease risk. Mol Cell Biol.

