# Supplementary material for: Alzheimer’s disease associated isoforms of human CD33 distinctively modulate microglial cell responses in 5XFAD mice
Source: Mol Neurodegener. 2024 May 27;19:42. doi: 10.1186/s13024-024-00734-8 (PMC11129479; doi:10.1186/s13024-024-00734-8)

Supporting Information

Alzheimer's disease associated isoforms of human CD33 distinctively modulate microglial cell responses in 5XFAD mice

Ghazaleh Eskandari-Sedighi[1], Madeline Crichton[1], Sameera Zia[2], Erik Gomez-Cardona[3], Leonardo M. Cortez[2,4,5], Zain H. Patel[6], Kei Takahashi-Yamashiro[1], Chris D. St. Laurent[1], Gaurav Sidhu[1], Susmita Sarkar[1], Vivian Aghanya[1], Valerie L. Sim[2,4,5], Qiumin Tan[6], Olivier Julien[3,5], Jason R. Plemel[2,5,7], Matthew S. Macauley[1,5,7,*]

[1] Department of Chemistry, University of Alberta, Edmonton, Canada.
[2] Division of Neurology, Department of Medicine, University of Alberta, Edmonton, Canada.
[3] Department of Biochemistry, University of Alberta, Edmonton, Canada.
[4] Centre for Prions and Protein Folding Diseases, University of Alberta, Edmonton, Canada.
[5] Neuroscience and Mental Health Institute, University of Alberta, Edmonton, Canada.
[6] Department of Cell Biology, University of Alberta, Edmonton, Canada.
[7] Department of Medical Microbiology and Immunology, University of Alberta, Edmonton, Canada.

[*]Corresponding author: macauley@ualberta.ca

Supplementary Figure 1: Control 5XFAD mice from CD33M or CD33m breeders show no significant differences in total Aβ levels. (a,b) Quantification of total Aβ levels in control 5XFAD mice obtained from either CD33M or CD33m breeders at 4 months **(a)** in pooled mice (n = 16 and 17, from CD33M and CD33m breeders, respectively) and **(b)** in sex separated mice (n = 9 females and 7 males from CD33M breeders and n=7 females and 10 males, from CD33m breeders). **(c,d)** Quantification of total Aβ levels at 8 months in **(c)** in pooled mice (n = 25 and 21 from CD33M and CD33m breeders respectively) and **(d)** in sex-separated mice (n = 14 females and 11 males from CD33M breeding and n=10 females and 11 males from CD33m breeders).

Supplementary Figure 2: Quantitative analysis of plaque load, microglia density, and plaque compaction in sex-separated mice. (a-d) Quantification of the total Aβ levels in female and male mice within the subiculum at **(a)** 4 months for females (n=20, 15, and 16 from control, CD33M and CD33m genotypes, respectively) and males (n= 20, 18, and 12 from control, CD33M and CD33m genotypes, respectively) and **(b)** at 8 months for females (n=22, 14, and 13 from control, CD33M and CD33m genotypes, respectively) and males (n=18, 11, and 10 from control, CD33M, and CD33m genotypes, respectively). **(e-j)** Female and male mice within each genotype show no significant differences in Iba1 density at **(e-g)** 4 months and **(h-j)** 8 months. For the 4 months analysis, n= 6, 6, and 9 females and n=6, 7, and 6 males from control, $CD33M^+$ and $CD33m^+$ mice, respectively. For the analysis at 8 months, n=16, 11, and 9 females and n= 10, 10, and 9 males from control, $CD33M^+$ and $CD33m^+$ mice, respectively.) **(k,l)** The genotype-driven effects of amyloid compaction (regional ThioS area/Aβ area) at 8 months (n=5 mice/genotype and per sex) **(m,n)** genotype-driven effects on ratio of $ThioS^+$ plaques at 8 months. (n=5 mice/genotype and per sex). **(o,p)** PAM density in female and male mice at 4 months (n=5 mice/genotype and per sex.

150 plaques per genotype were quantified). **(q,r)** PAM density in female and male mice at 8 months (n=5 mice/genotype and per sex. 150 plaques per genotype were quantified).

Supplementary Figure 3: Region-specific analyses of Aβ levels at 4 and 8 months. Quantification of total Aβ levels in the **(a)** hippocampus and **(b)** cortex at 4 months for pooled males (squares; n = 7, 5, and 7 for control, CD33M, and CD33m, respectively) and females (circles; n = 10, 5, and 7 for control, CD33M, and CD33m, respectively). **(c,d)** Quantification of total Aβ levels in the **(c)** hippocampus and **(d)** cortex at 8 months for pooled males (squares; n = 7, 5, and 7 for control, CD33M, and CD33m, respectively) and females (circles; n = 10, 5, and 7 for control, CD33M, and CD33m, respectively). **(e-f)** Quantification of total number of Aβ deposits in control, CD33M, and CD33m mice at **(e)** 4 and **(f)** 8 months, stained with an anti-Aβ antibody (n = 5 male and 5 female mice per genotype). Automated counting was done by Zeiss analysis software, and the total number of assemblies with a diameter larger than 20 µm were quantified.

Supplementary Figure 4: Quantitative analysis of plaque compaction. a) Quantification of the total Aβ level, **b)** ThioS level, and **c)** Compaction in the dorsal subiculum region. Data was quantified from 5 males and 5 females per genotype. **(d-g)** Individual Aβ plaques were quantified for total area staining of **(d)** anti-Aβ and **(e)** ThioS, from 5XFAD mice at 4 months. Individual Aβ plaques were quantified for area staining of **(f)** anti-Aβ and **(g)** ThioS, from control, CD33M^{+}, and CD33m^{+} 5XFAD mice at 8 months. For both analyses, a total of 300 plaques per mouse, from 5 males and 5 females were analyzed. **h)** The total number of Aβ deposits (MOAB) with a diameter larger than 20 µM were quantified from half brain images of 5 male and 5 female mice per

genotype. The percentage of Aβ deposits with **(i)** or without **(j)** ThioS core were quantified in the same images. **k)** Representative images of ThioS plaque core with different levels of sphericity. Scale bar = 20 μm**. l)** Quantitative analysis of ThioS core sphericity on control, $CD33M^{+}$, and $CD33m^{+}$ 5XFAD mice at 8 months. A total of 300 plaques per mouse, from 5 males and 5 females were analyzed.

Supplementary Figure 5: Biochemical analysis of soluble and insoluble Aβ fractions at 8 months. a-c) Quantitative analysis of soluble fraction of brain for **(a)** $A\beta_{1-42}$, **(b)** $A\beta_{1-38}$, and **(c)** $A\beta_{1-40}$ of control, CD33M and CD33m mice at 8 months. An equal number of male and female mice (n=5-6 per sex) were analyzed per genotype. **(d-f)** Quantitative analysis of insoluble fraction for **(d)** $A\beta_{1-42}$, **(e)** $A\beta_{1-38}$, and **(f)** $A\beta_{1-40}$ of control, CD33M and CD33m mice at 8 months. An equal number of male and female mice (n=5-6 per sex) were analyzed per genotype. **g)** $A\beta_{1-42}$/ $A\beta_{1-40}$ ratio in the insoluble fraction of control, CD33M and CD33m mice at 8 months. **h)** Quantitative analysis of human oligomeric $A\beta_{1-42}$ in the soluble fraction of brain homogenate of control, CD33M and CD33m mice at 8 months. **i)** The insoluble fraction of 4 control mice were subjected to a PK gradient of 0-120 μg/ml, to identify the minimum PK concentration that induces highest drop in Aβ levels. A PK concentration of 20 μg/ml was selected for further analysis of all samples.

Supplementary Figure 6: Quantitative analysis of ApoE protein levels in the soluble and insoluble fraction of brain. The ApoE protein levels in the soluble **(a)** and **(b)** insoluble fractions of brain from control, $CD33M^{+}$ and $CD33m^{+}$ 5XFAD mice were quantified by ELISA. **a)** No significant differences were observed in the ApoE levels of the soluble fractions. b) The $CD33m^{+}$

brains had significantly higher levels of ApoE in the insoluble fraction compared to control mice. A total n=10 mice per group were analyzed (5 male and 5 female).

Supplementary Figure 7: CD33m^{+} microglia have increased internalized Aβ in CD68^{+} structures within microglia. a) Representative close-up images of CD68^{+} structures (yellow) inside microglia (Iba1, red) interacting/associated with Aβ (MOAB, white). Scale bar = 10 µm. **b)** Representative confocal fluorescent images of internalized Aβ from control, CD33M^{+}, or CD33m^{+} 5XFAD mice at 8 months. IF images are co-stained with anti-Aβ antibody (white), anti-Iba1 (red), and Hoechst (blue). Scale bar = 20 µm. **c)** Quantification of internalized Aβ measured as the area of Aβ signal overlapping within Iba1 A total of 300 plaques from 5 male and 5 female mice were quantified for each group.

Supplementary Figure 8: Quantitative analyses on plaque-microglia interface and plaque-associated microglia density: a) Quantification of plaque associated microglia normalized to µm^2 of plaque area at 4 months. A total of 200 plaques in subiculum of 10 mice (5 males and 5 females per group) were analyzed **b)** Quantification of plaque-microglia interface in 5XFAD mice at 4 months, measured as the percentage area of ThioS perimeter with overlapping Iba1 signal. A total of 200 plaques in the subiculum of 10 mice (5 males and 5 females per group) were analyzed.

Supplementary Figure 9: Iba1 density of 5XFAD mice expressing hCD33 isoforms at 4 and 8 months. a) Representative epifluorescent images in 5XFAD mice at 8 months by IF imaging with the anti-Iba1 antibody (red) and DAPI (blue). Scale bar = 1000 µm. **b)** Quantification of total Iba1 density at 8 months for pooled male (squares; n = 10, 8, and 8 for control, CD33M, and

CD33m, respectively) and female (circles; n = 9, 9, and 7 for control, CD33M, and CD33m, respectively) mice. **c)** Representative epifluorescent images of in 5XFAD mice at 4 months by IF imaging with the anti-Iba1 antibody (red) and DAPI (blue). **d)** Quantification of total Iba1 density at 4 months for pooled male (squares; n = 7, 6, and 6 for control, CD33M, and CD33m, respectively) and female (circles; n = 5, 5, and 5 for control, CD33M, and CD33m, respectively) mice.

Supplementary Figure 10: Differentially expressed proteins from the mass spectrometry proteomics profile. Volcano plots presenting whole brain proteome comparison between **(a)** CD33M versus control, **(b)** CD33m versus control, **(c)** CD33m versus CD33M, and **(d)** non-FAD control versus CD33m. The boxes indicate the proteins significantly increased (red box) and decreased (blue box), with a cut-off value of 1.5-fold change and a significant cut off value of $P<0.05$. The arrow in (**b**) points to upregulation of the protein nestin in the brain of CD33m mice. The yellow dots in **(d**) corresponds to proteins that presented significant changes amongst 5XFAD groups.

Supplementary Figure 11. Total protein levels of ABCG2 and FABP7 in whole brain proteome. The two proteins that are highly expressed by neural stem cell markers and were detectable in the proteomics, ABCG2 (**a**) and FABP7 (**b**) show no significant differences in abundance between genotypes.

Supplementary Figure 12: Gene ontology enrichment of the candidate proteins identified in brain proteome analysis. Altered biological processes from significant proteins identified per proteomic comparison are presented. GO analysis for the selected candidate protein hits was performed and over- or underrepresented GO entries with *p*-value$\leq$ 0.05 were considered significant.

Supplementary Figure 13: Cell-specific abundance of proteomics hits. Heatmap chart of identified proteins with cell-type specific expression patterns. The expression levels of each protein were revised from the mouse protein atlas, their average abundance was normalized (z-score). Proteins with significantly different abundance have been bolded.

Supplementary Figure 14: Batch integration of scRNAseq data. Three different integration methods were applied on our dataset to correct for possible batch effects. **(a-d)** original Merged data **(a)**, Harmony (v0.1.0) **(b)**, Seurat Integration **(c)**, and FastMNN **(d)** all preserve the major identified clusters and show minimal differences.

Supplementary Figure 15: Mouse and human CD33 transcript levels in the microglia of 5XFAD mice. **a)** *hCD33* transcript levels in the microglia of Control 5XFAD, CD33M^{+} 5XFAD, and CD33m^{+} 5XFAD mice. All datasets are derived from aligning our scRNAseq datasets with the inclusion of *hCD33* sequence. This method cannot be used to differentiate CD33M and CD33m transcripts due to extensive overlap between the two isoforms. **b)** *mCd33* transcript levels in the microglia of Control 5XFAD, CD33M^{+} 5XFAD, and CD33m^{+} 5XFAD mice.

Supplementary Figure 16: Pseudobulk comparisons and GO enrichment of DEGs further supports enhanced activation in CD33m$^+$ microglia. a) Volcano plot showing the differentially expressed genes between pairs of libraires of CD33M versus CD33m. **b)** Alluvial plot linking microglial clusters from all genotypes to gene ontology terms. **c)** violin plots presenting gene expression level of DAM associated genes in all three genotypes on the 5XFAD background. **d)** volcano plots presenting *Cspg4* and *Vim* gene expression levels in all three genotypes on the 5XFAD background. The *p* values<0.0001 are presented by four starts.

Supplementary Figure 17: Immunofluorescent analysis of Clec7a levels in 5XFAD mice at 8 months. a) Representative images of Clec7a staining (yellow) in microglia (Iba1, red) surrounding ThioS plaques (blue). Scale bar = 20 μm **b)** Quantitative analysis of Clec7a levels relative to Iba1 per FOV in control, CD33M$^+$, and CD33m$^+$ 5XFAD mice at 8 months. A total of 8 images from dorsal subiculum of each mouse was analyzed (n=5/genotype).

Supplementary Figure 18: Correlation of PAM and dystrophic neurites. a) Representative images of neuritic plaques stained with LAMP1 (yellow), co-stained with ThioS (blue) and Iba1 (red). CD33m$^+$ mice show a higher number of PAM and lower area of LAMP1. Scale bar = 50 μm. **b)** Quantification of absolute number of PAM associated with individual plaques in control, CD33M$^+$, and CD33m$^+$ 5XFAD mice at 8 months. A total of 300 plaques per mouse, from 5 males and 5 females per genotype were analyzed. **c)** Quantification of percentage of diffuse versus compact plaques within dystrophic neurites of control, CD33M$^+$, and CD33m$^+$ 5XFAD mice. A minimum of 15 FOV obtained from dorsal subiculum and frontal cortex adjacent to subiculum of 5 males and 5 females per genotype were analyzed. **d-f)** Correlation graph on the relationship

between size of dystrophic neurites and Iba1 density within subiculum of **(d)** control, **(e)** CD33M$^+$, and **(f)** CD33m$^+$ 5XFAD mice. A total of 17 FOV per genotype from 5 males and 5 females were analyzed. **g-j)** Correlation graph on the relationship between the size of dystrophic neurites and Aβ level within individual plaques in the subiculum of **(g)** control, **(h)** CD33M$^+$, **(i)** CD33m$^+$ 5XFAD mice, as well as **(j)** pooled mice from all genotypes. A total of 100 dystrophic neurites from 5 females and 5 males per genotype were analyzed.

Supplementary Figure 19: Additional parameters measured in the behavioral assays. (a-d) Results from the open-field test (OFT) carried out on control, CD33M$^+$, and CD33m$^+$ 5XFAD mice at 1 year (n=13, 4, and 10 female mice per control, CD33M, and CD33m groups, respectively). Quantification of **(a)** the time mice spent in the center of the field, **(b)** total distance travelled, **(c)** average velocity in the whole arena, and **(d)** average velocity in the center. **(e-h)** Results from the open-field assay carried out on control and CD33m$^+$ 5XFAD mice at 8 months of age (n=13 female and 7 male mice for the control group, and n=11 female and 10 male mice for the CD33m group were used). Quantification of **(e)** the time mice spent in the center of the field, **(f)** total distance travelled, **(g)** average velocity in the whole arena, and (h) average velocity in the center. **(i-l)** Sex separated analyses on light−dark box assay **(i,j)** and Y-maze **(k,l)** points to significant differences in male mice.

Suppl. Figure 1

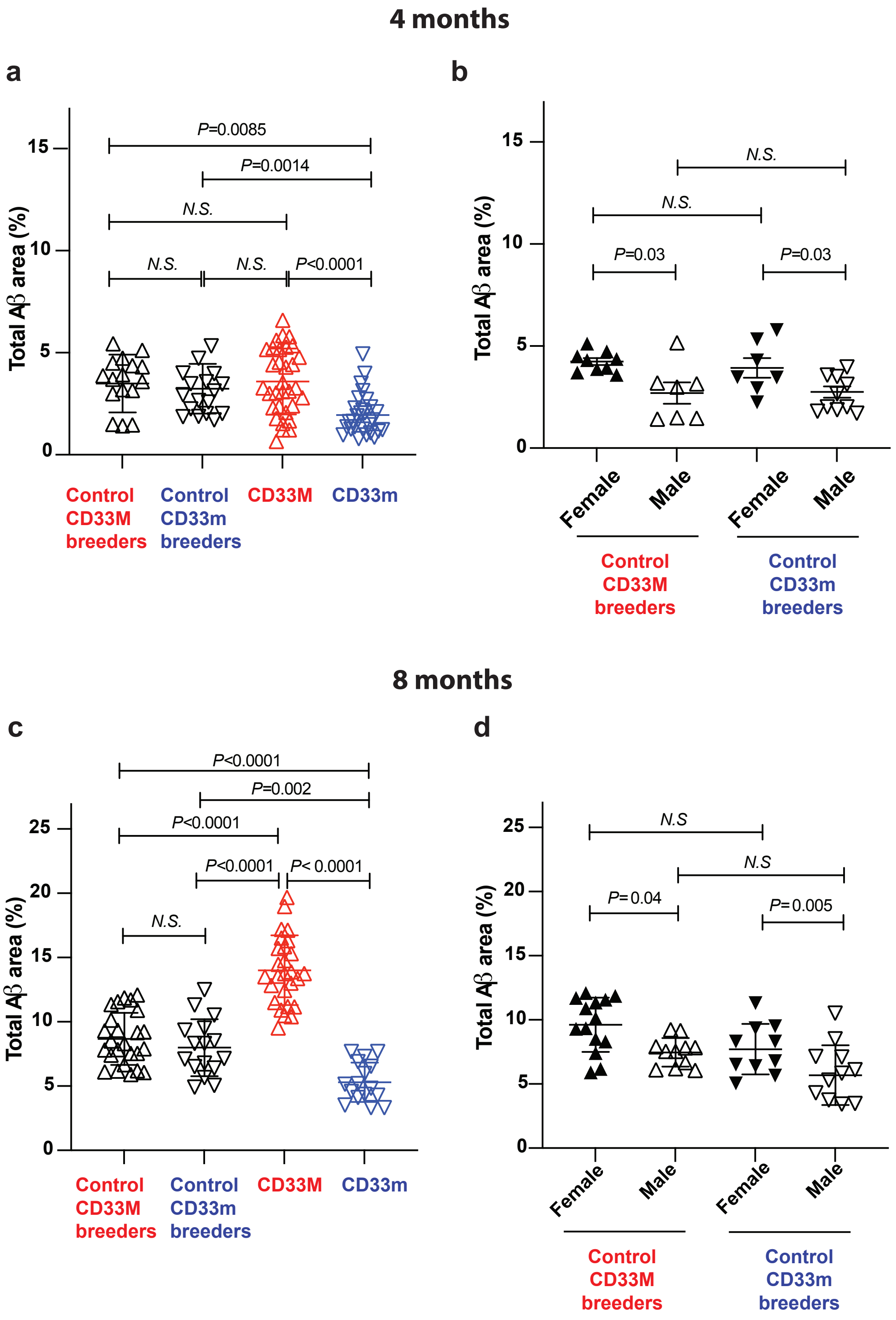

Suppl. Figure 2

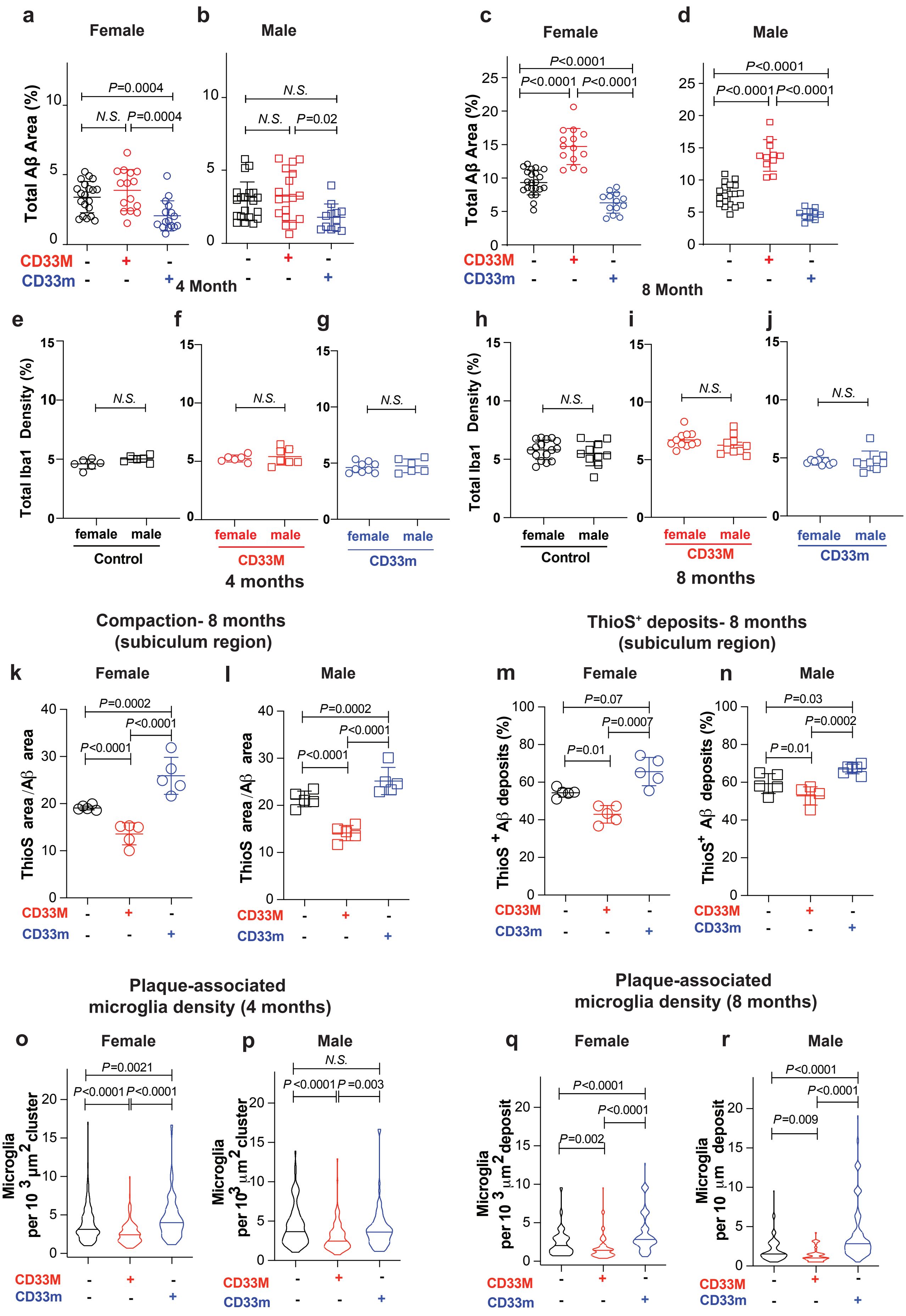

Suppl. Figure 3

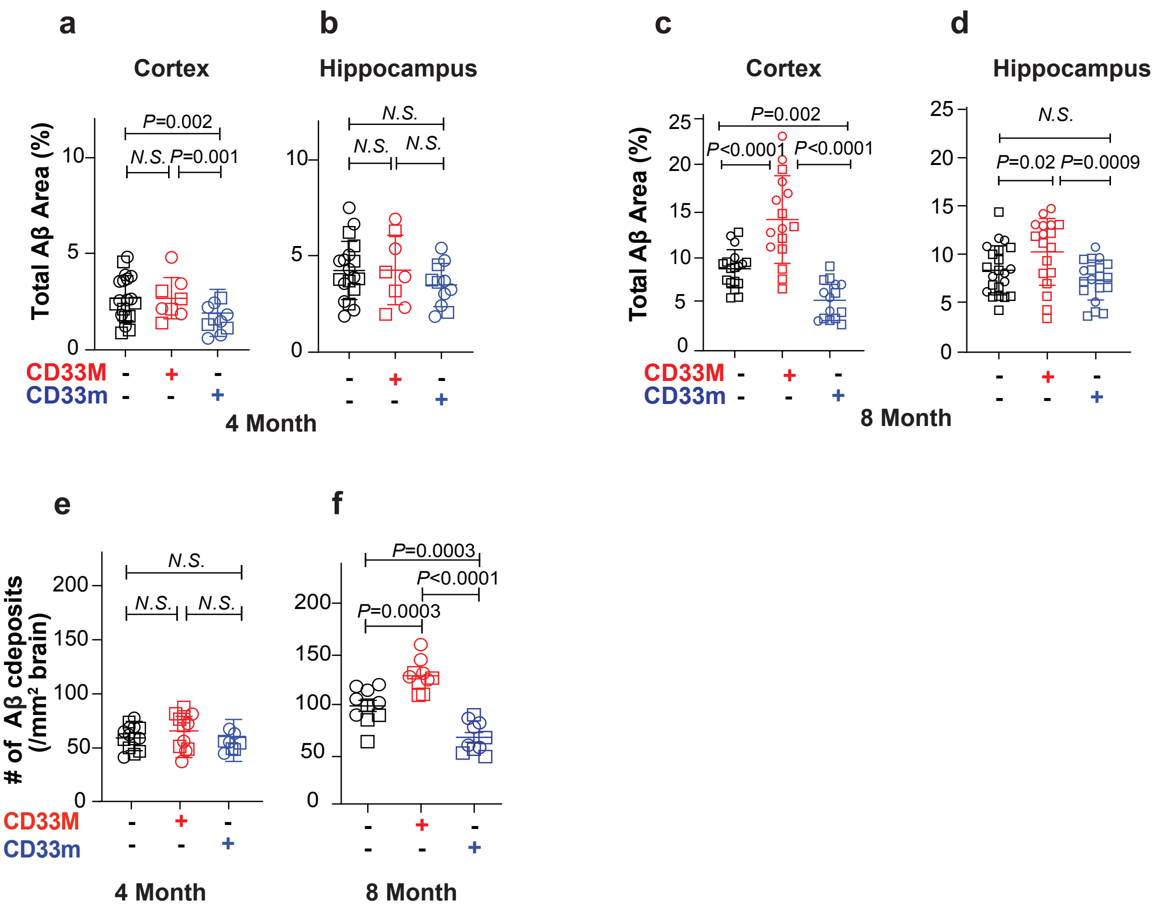

Suppl. Figure 4

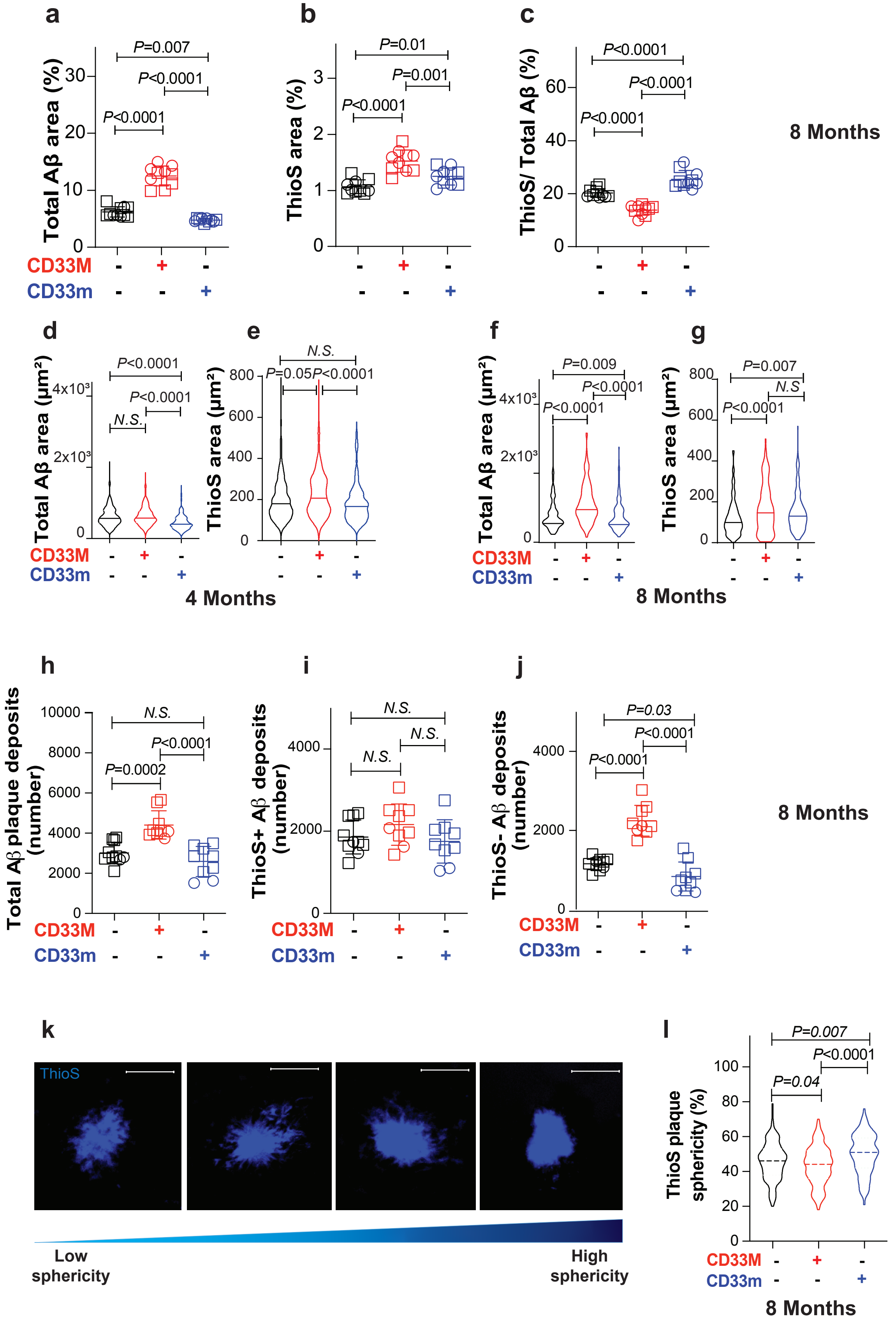

Suppl. Figure 5

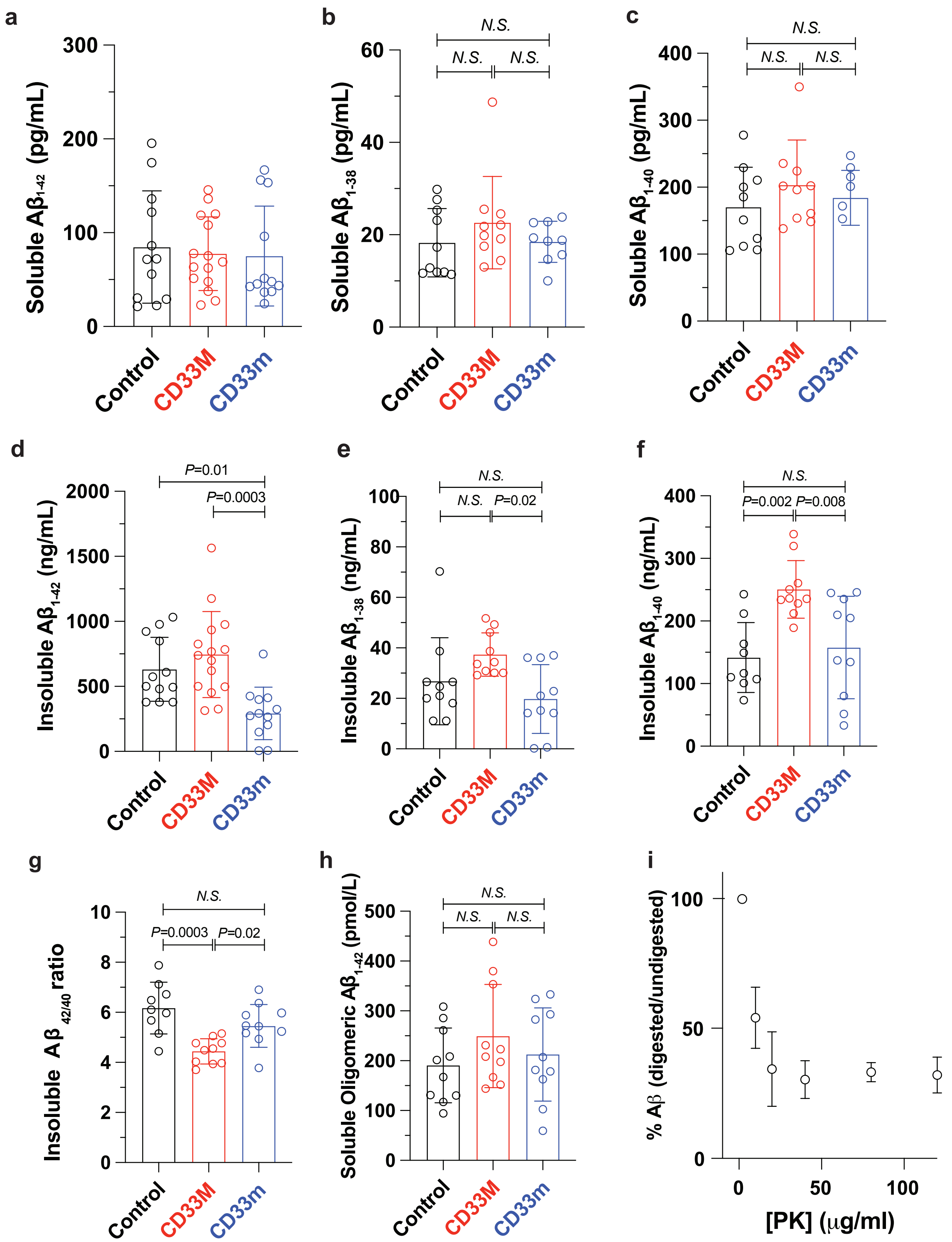

Suppl. Figure 6

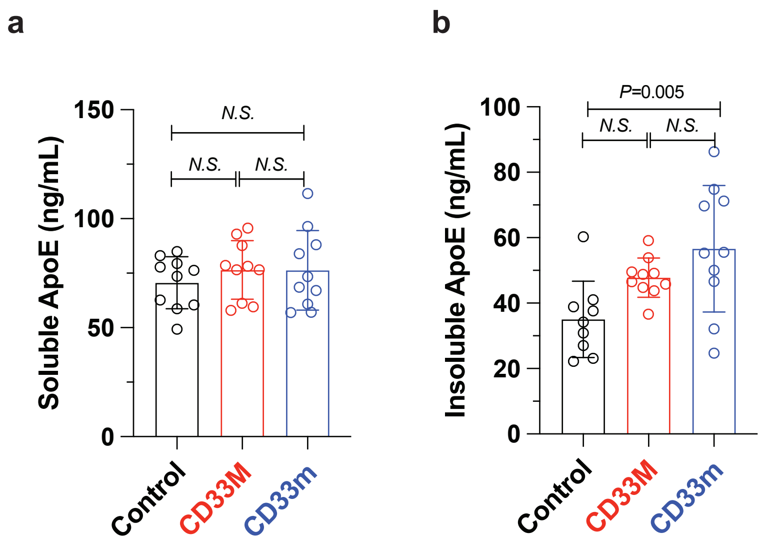

Suppl. Figure 7

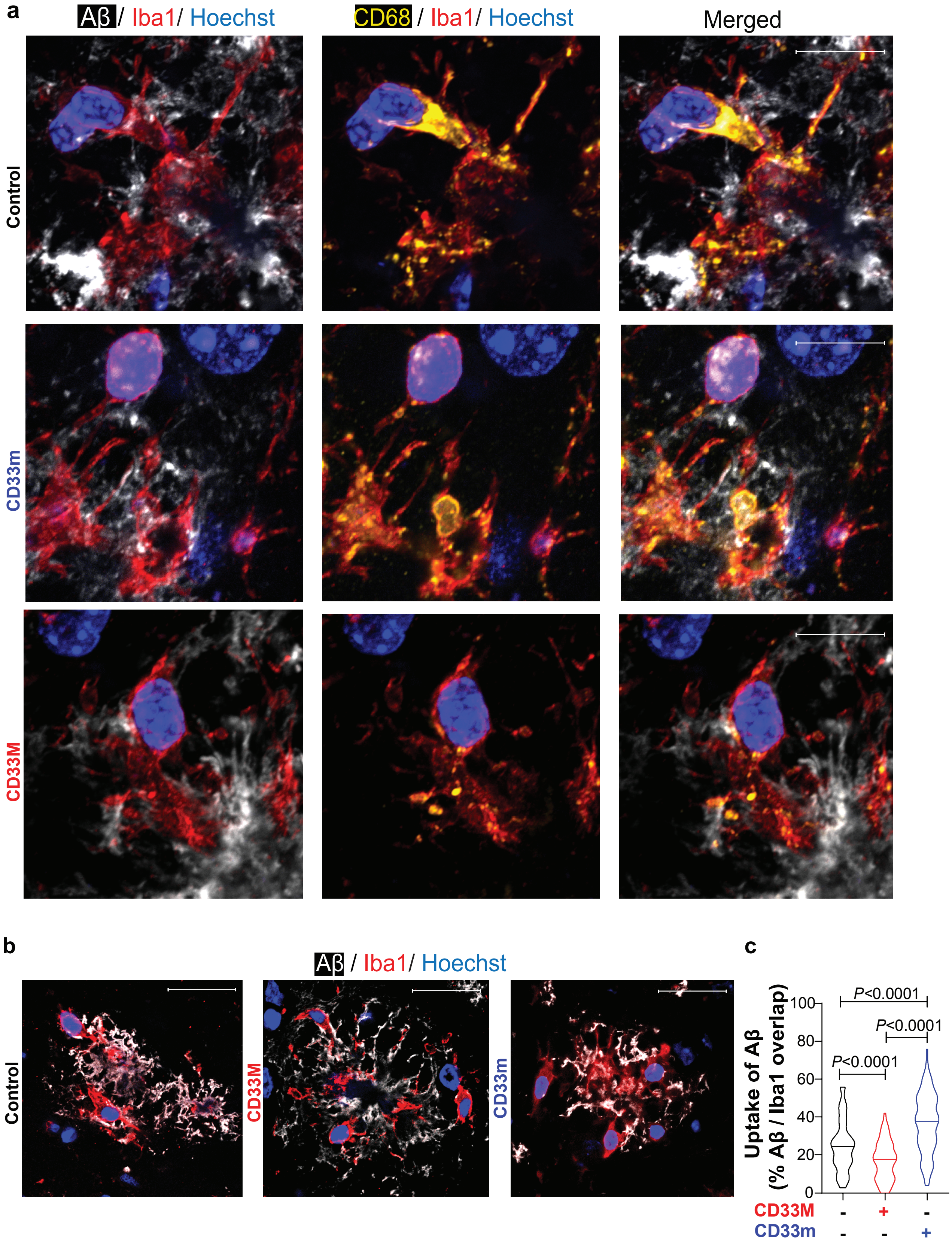

Suppl. Figure 8

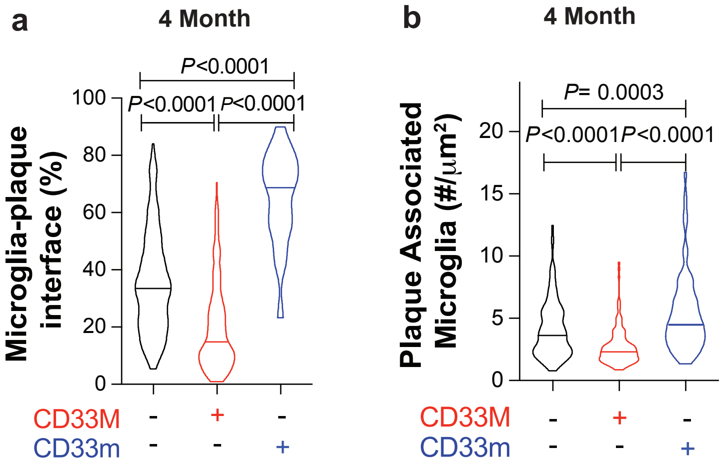

Suppl. Figure 9

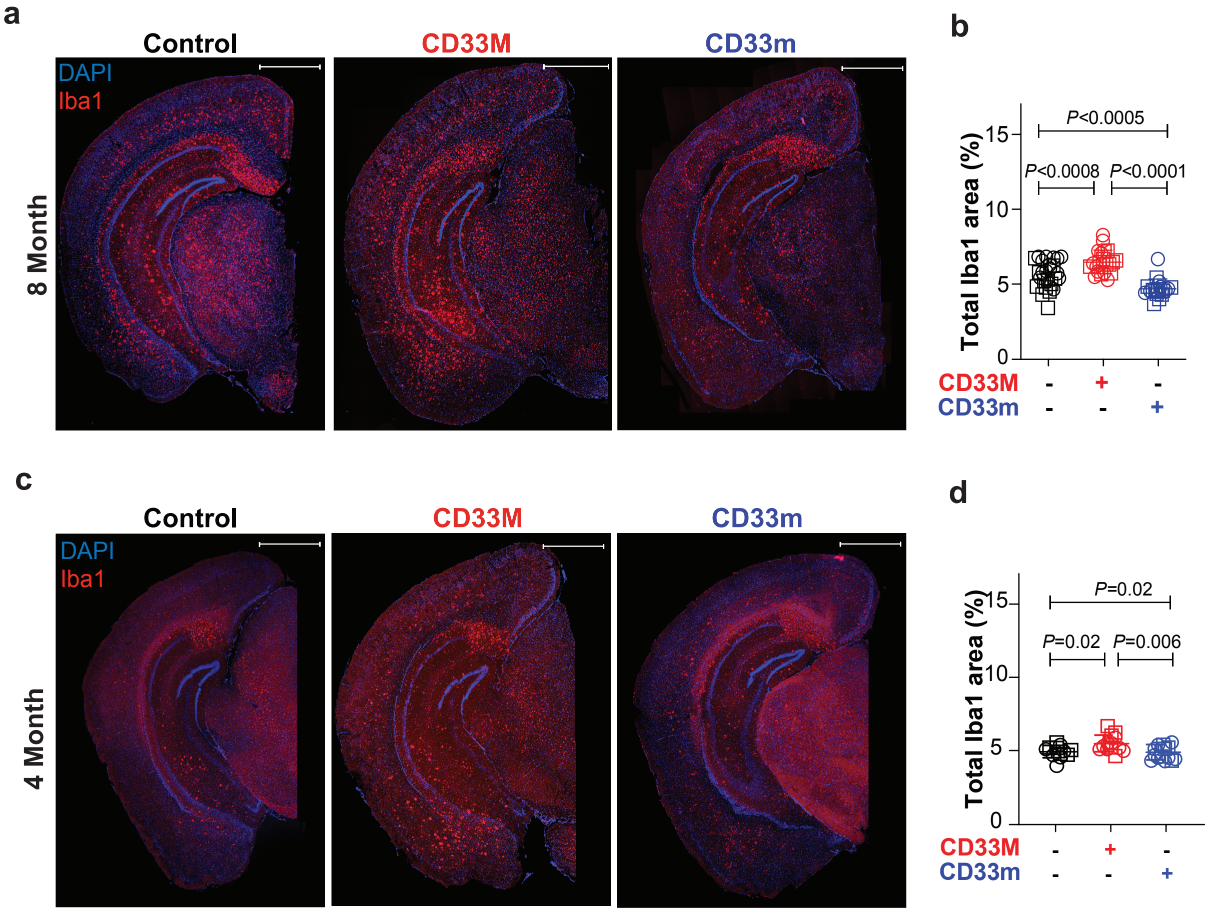

Suppl. Figure 10

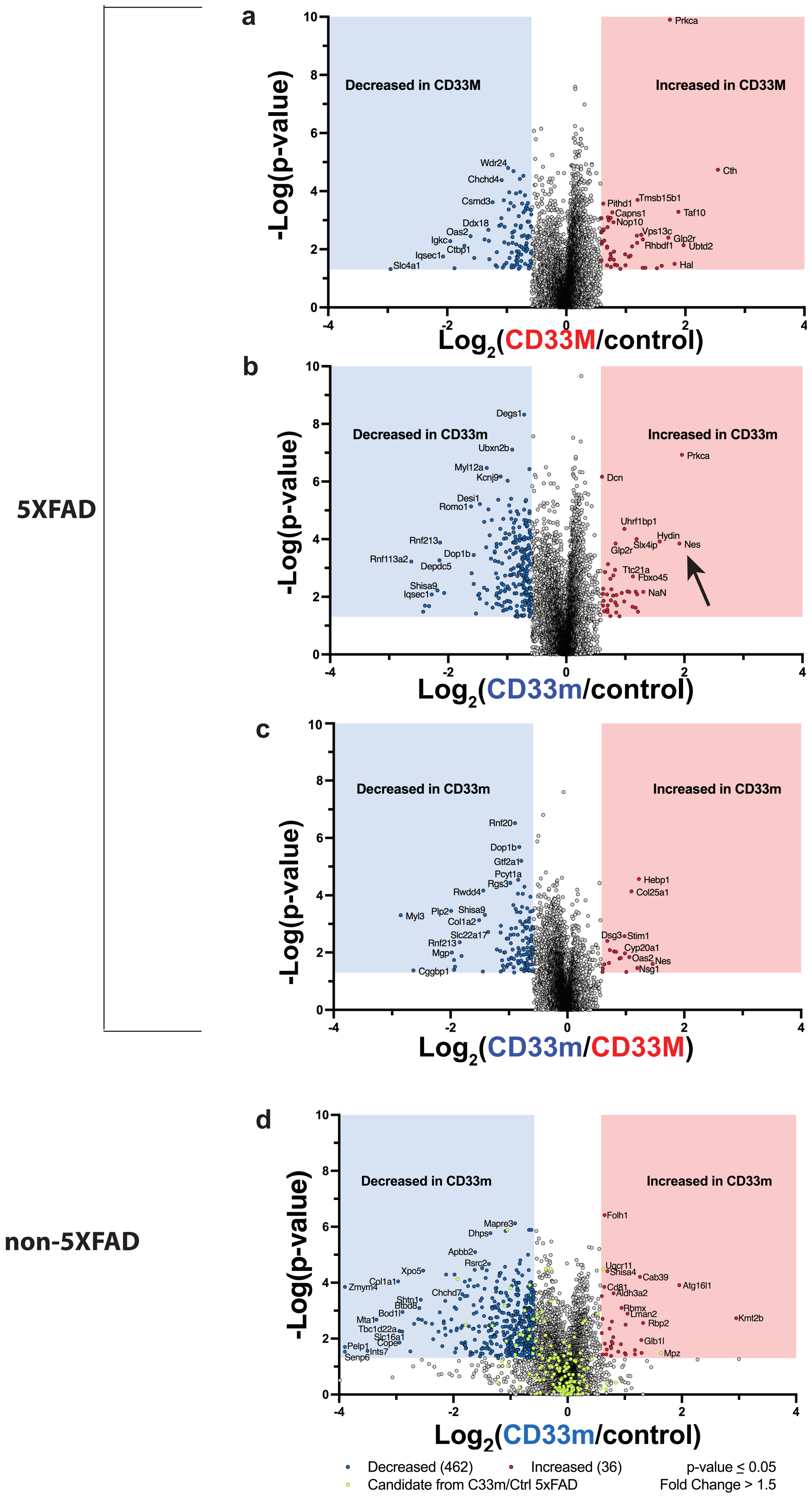

Suppl. Figure 11

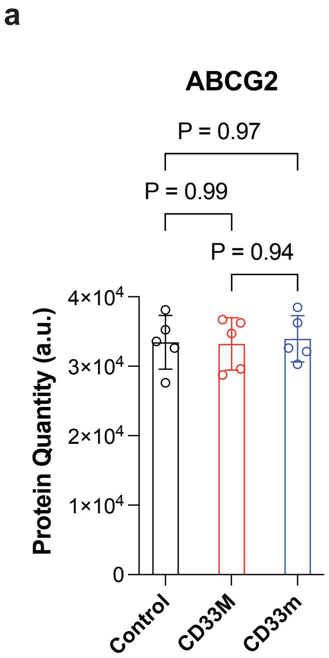

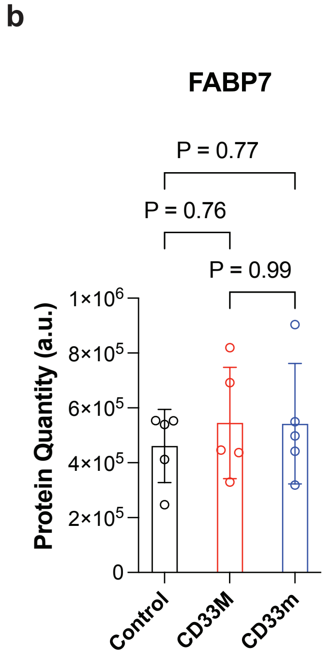

Suppl. Figure 12

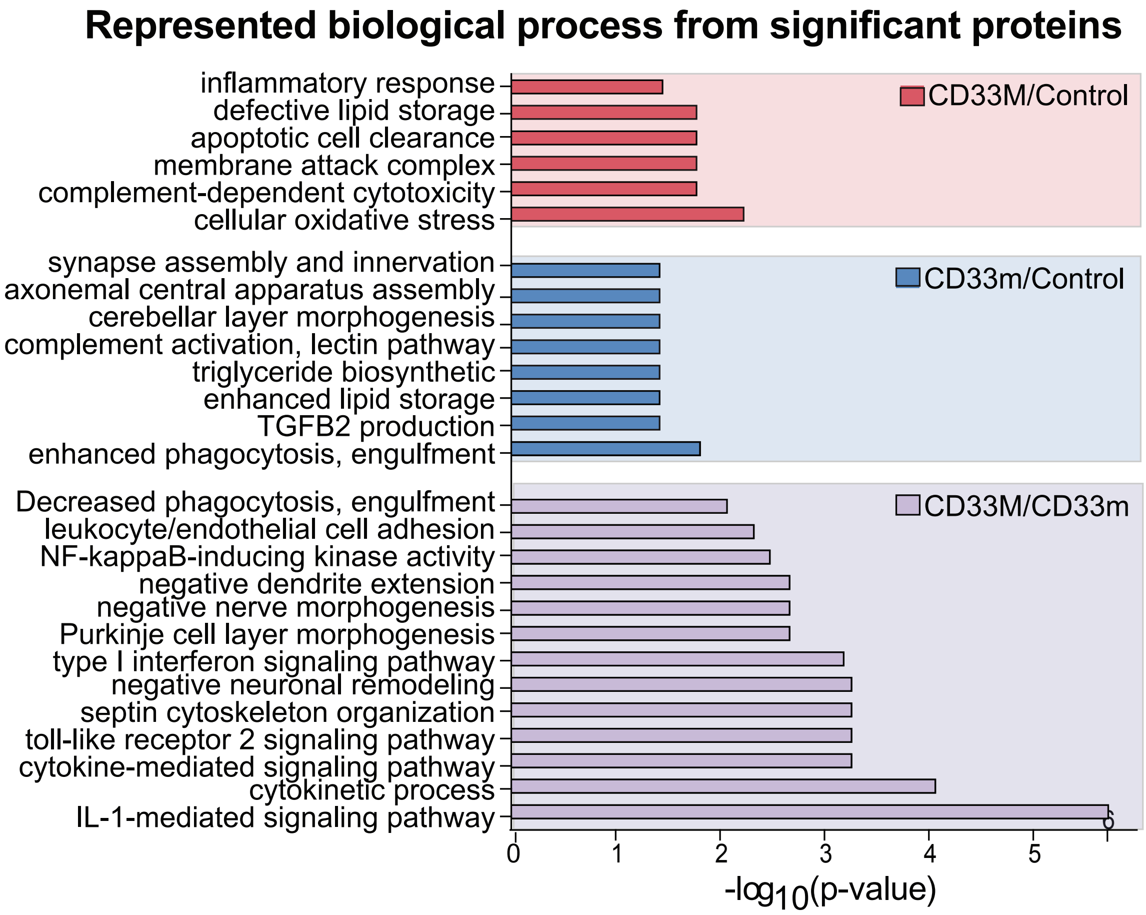

Suppl. Figure 13

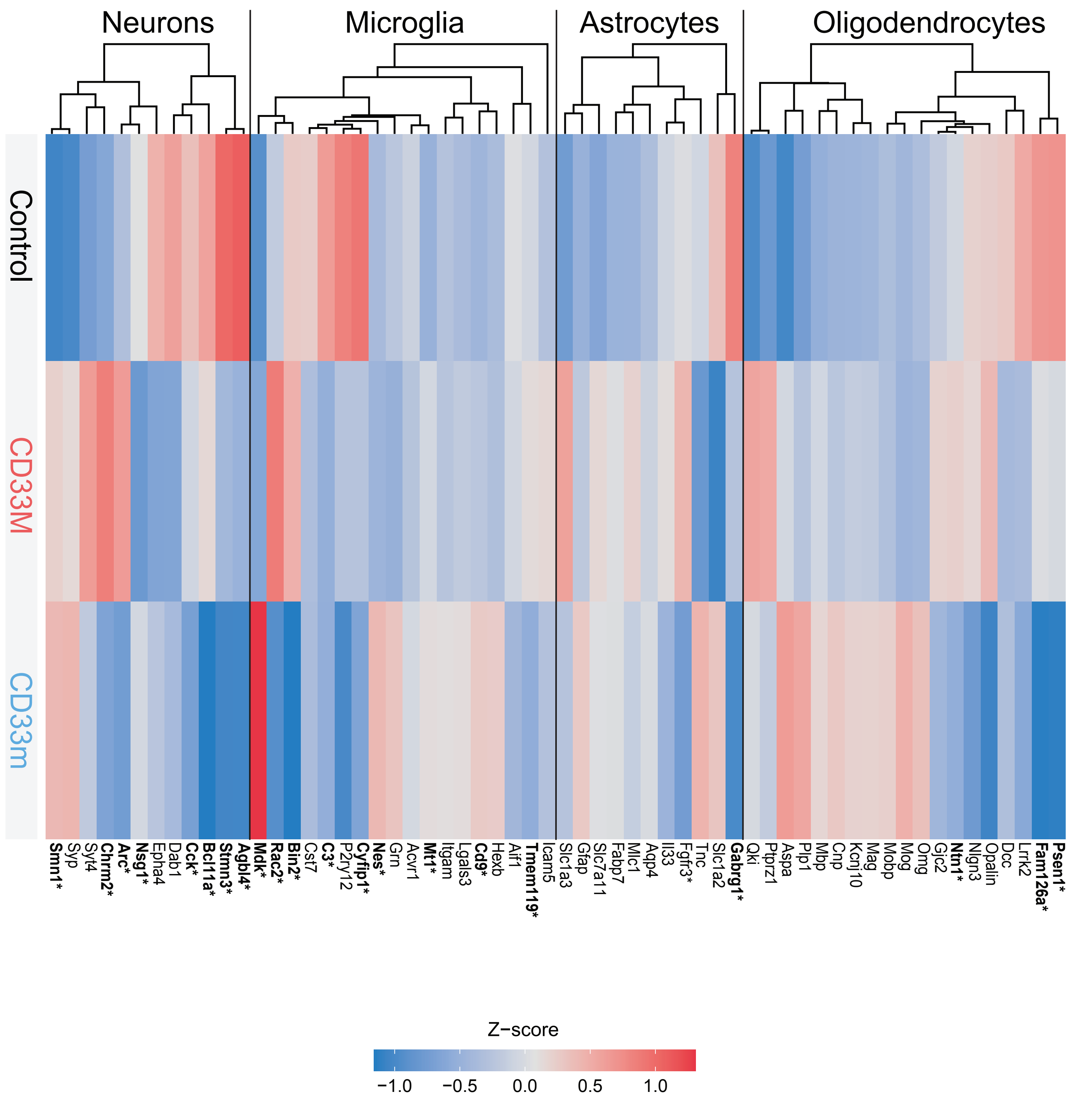

Suppl. Figure 14

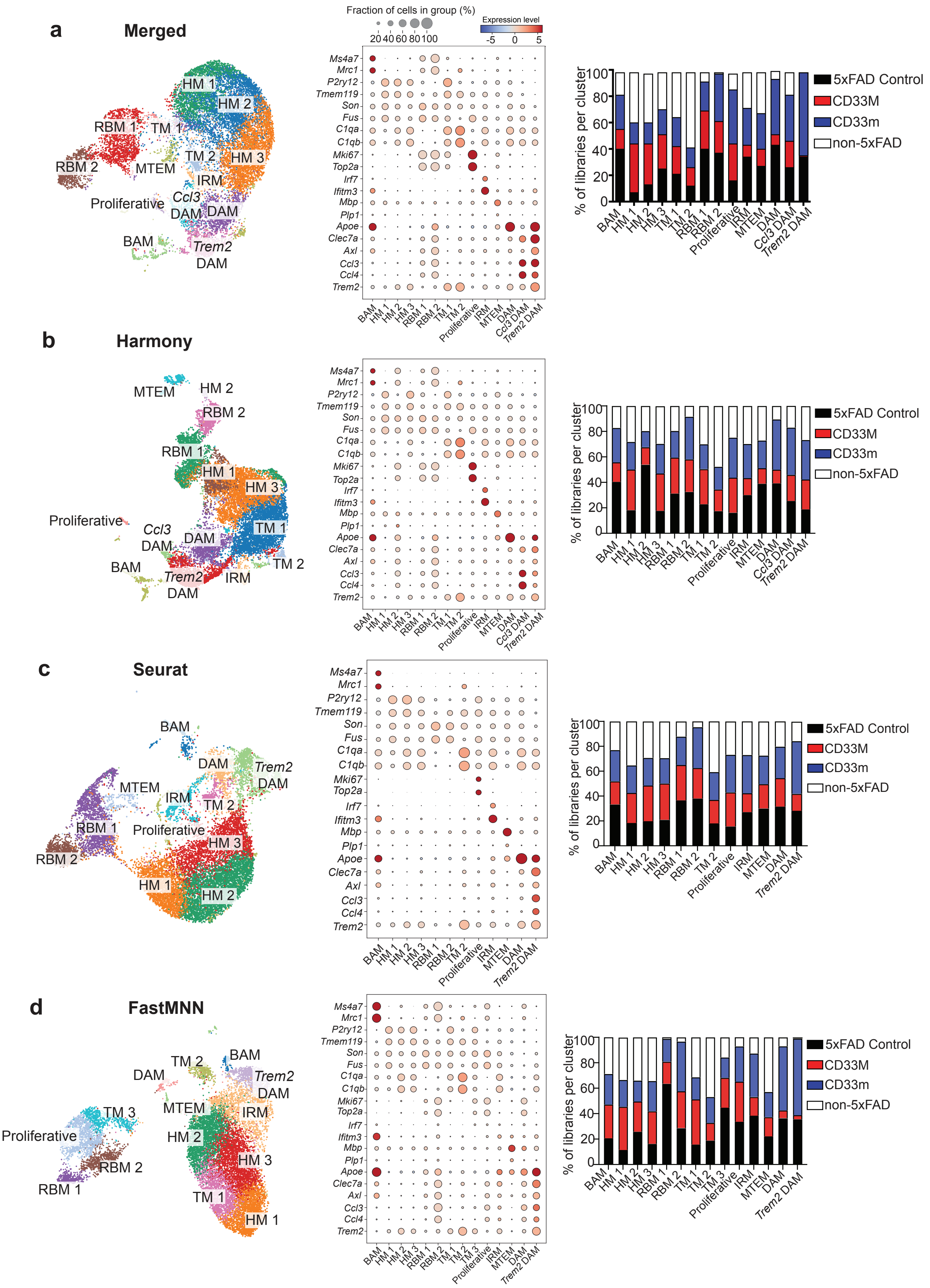

Suppl. Figure 15

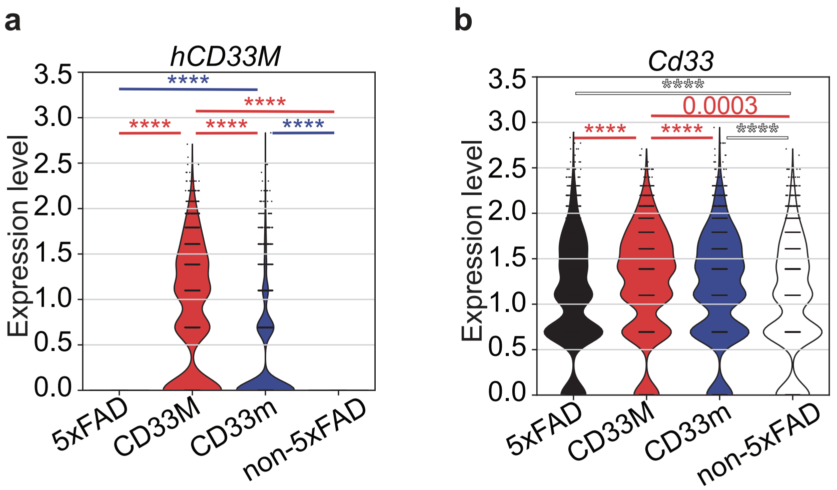

Suppl. Figure 16

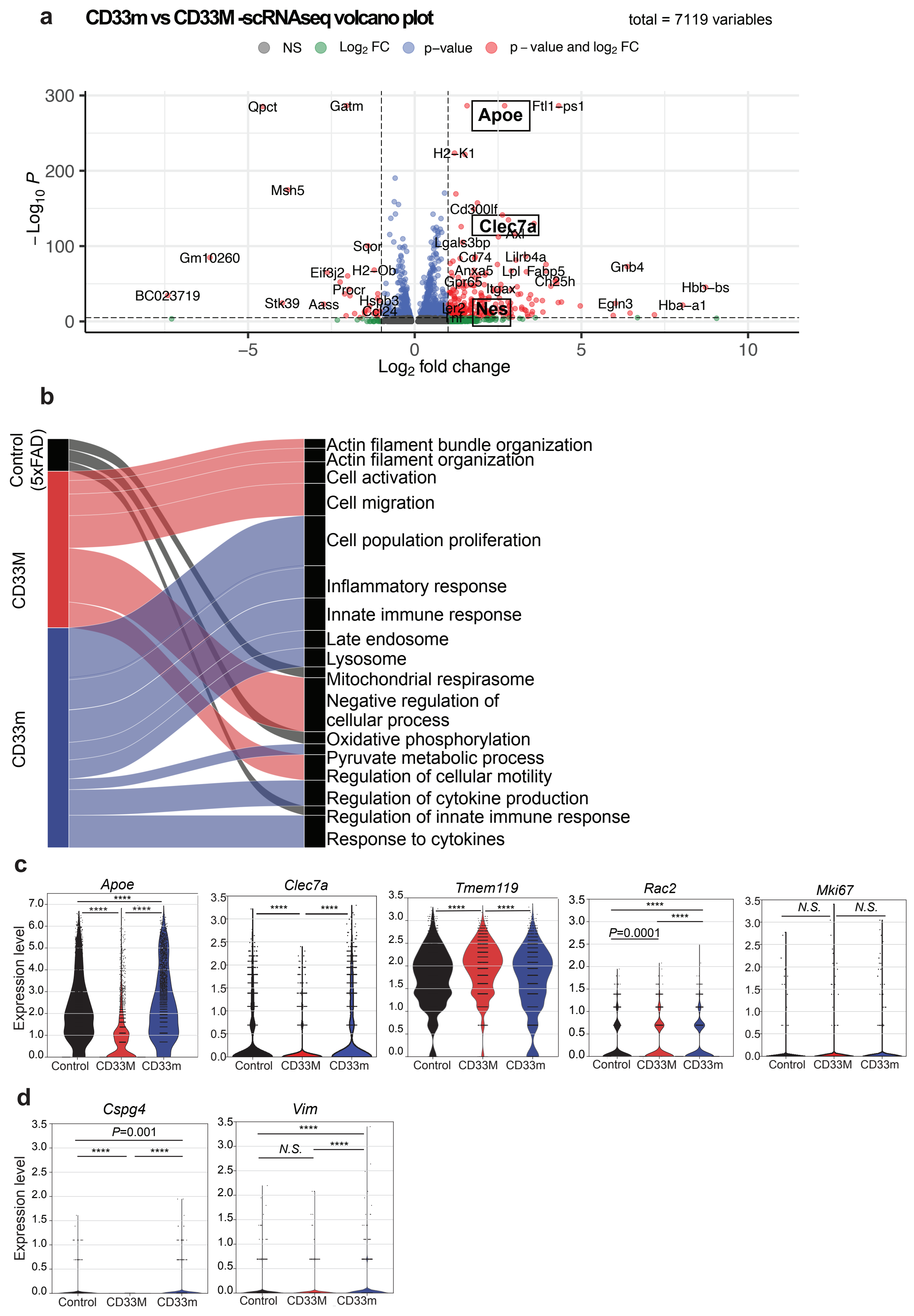

Suppl. Figure 17

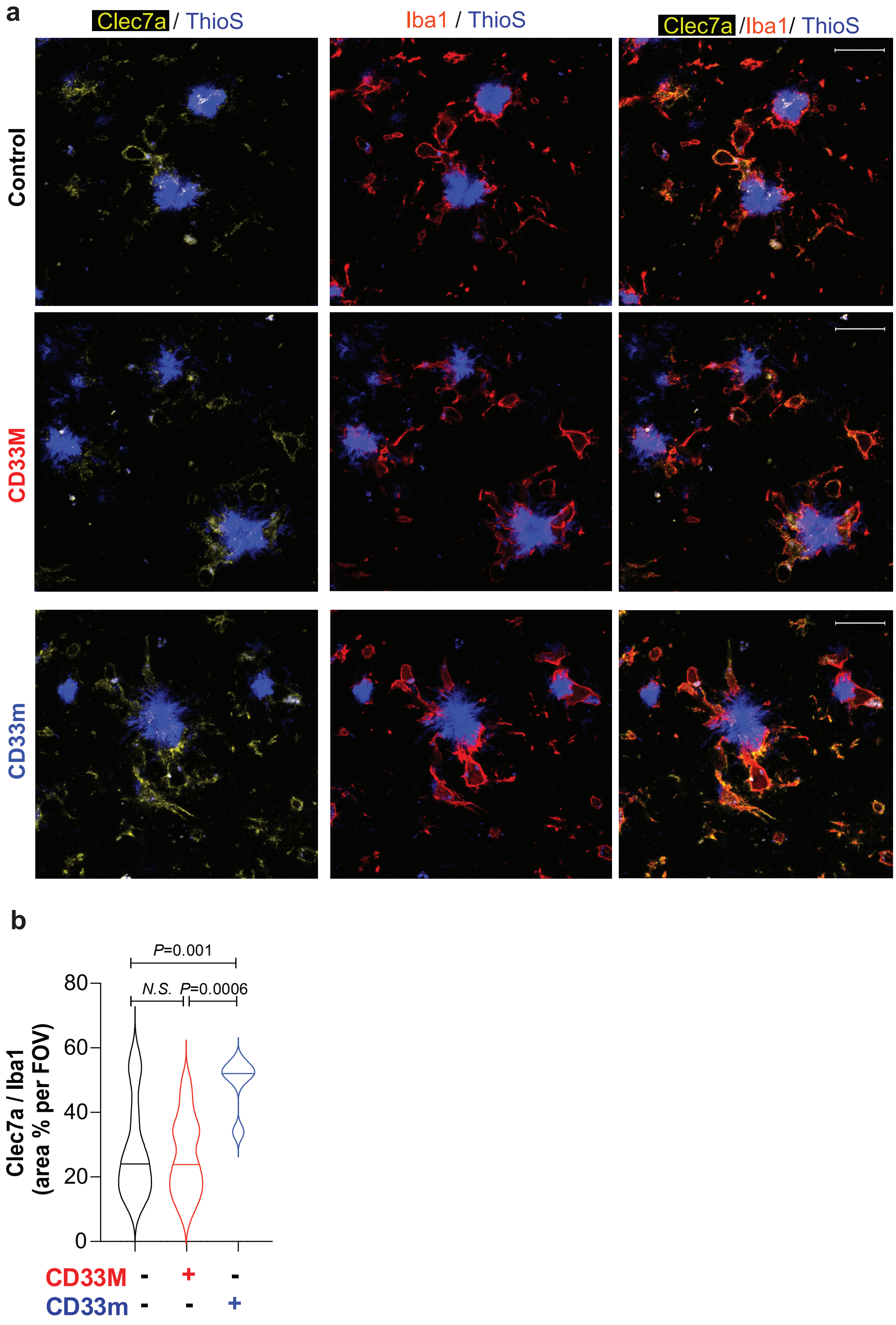

Suppl. Figure 18

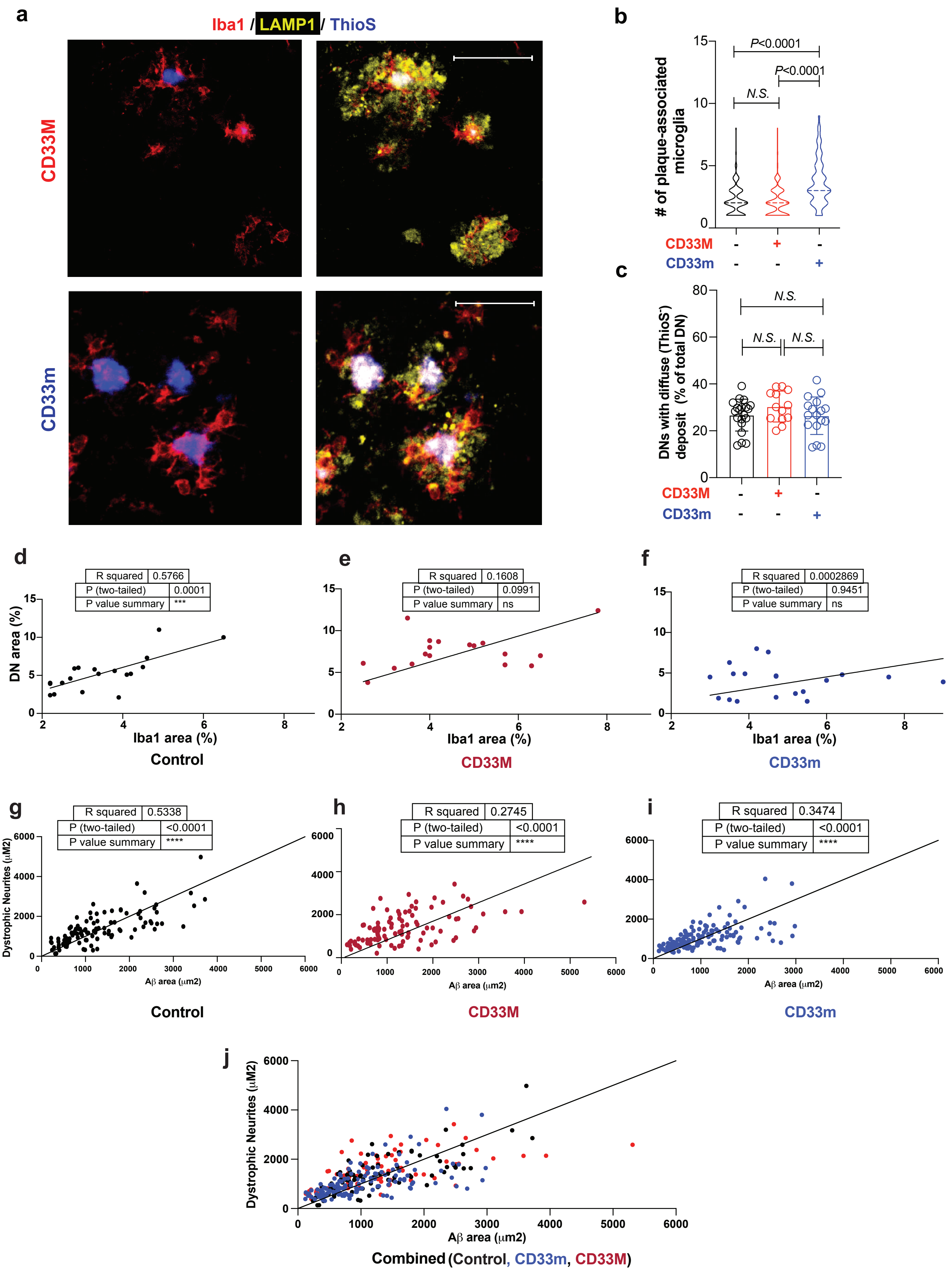

Suppl. Figure 19

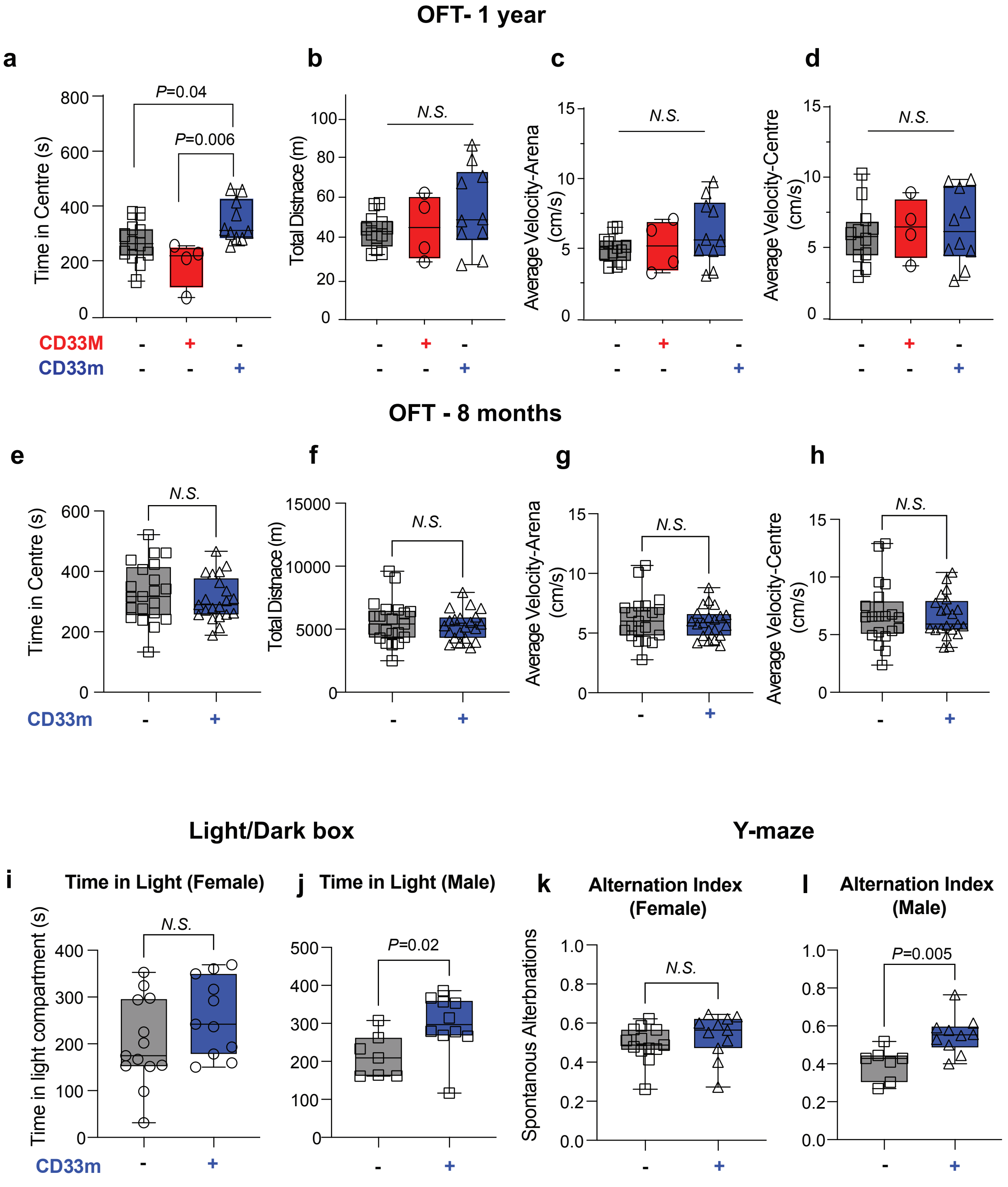

Supplement: Supplementary file 1 — Supplementary Material 1. [file 13024_2024_734_MOESM1_ESM.pdf]
